# Additively manufactured nano-mechanical energy harvesting systems: advancements, potential applications, challenges and future perspectives

**DOI:** 10.1186/s40580-021-00289-0

**Published:** 2021-12-01

**Authors:** Ammar Ahmed, Ali Azam, Yanen Wang, Zutao Zhang, Ning Li, Changyuan Jia, Ray Tahir Mushtaq, Mudassar Rehman, Thierno Gueye, Muhammad Bilal Shahid, Basit Ali Wajid

**Affiliations:** 1 Department of Industry Engineering, Northwestern Polytechnical University, Xi’an, 710072 People’s Republic of China; 2 School of Mechanical Engineering, Southwest Jiaotong University, Chengdu, 610031 People’s Republic of China; 3 Graduate School of Tangshan, Southwest Jiaotong University, Tangshan, 063008 People’s Republic of China; 4 School of Electrical Engineering, Southwest Jiaotong University, Chengdu, 610031 People’s Republic of China; 5 School of Mechanical Engineering, Xi’an Jiaotong University, Xi’an, People’s Republic of China; 6 Mechanical Engineering Department, University of Engineering and Technology Lahore, Lahore, Pakistan

**Keywords:** Nano-energy, Energy harvesting, Renewable energy, Sustainability, 3D printing, Challenges

## Abstract

Additively manufactured nano-MEH systems are widely used to harvest energy from renewable and sustainable energy sources such as wind, ocean, sunlight, raindrops, and ambient vibrations. A comprehensive study focusing on in-depth technology evolution, applications, problems, and future trends of specifically 3D printed nano-MEH systems with an energy point of view is rarely conducted. Therefore, this paper looks into the state-of-the-art technologies, energy harvesting sources/methods, performance, implementations, emerging applications, potential challenges, and future perspectives of additively manufactured nano-mechanical energy harvesting (3DP-NMEH) systems. The prevailing challenges concerning renewable energy harvesting capacities, optimal energy scavenging, power management, material functionalization, sustainable prototyping strategies, new materials, commercialization, and hybridization are discussed. A novel solution is proposed for renewable energy generation and medicinal purposes based on the sustainable utilization of recyclable municipal and medical waste generated during the COVID-19 pandemic. Finally, recommendations for future research are presented concerning the cutting-edge issues hurdling the optimal exploitation of renewable energy resources through NMEHs. China and the USA are the most significant leading forces in enhancing 3DP-NMEH technology, with more than 75% contributions collectively. The reported output energy capacities of additively manufactured nano-MEH systems were 0.5–32 mW, 0.0002–45.6 mW, and 0.3–4.67 mW for electromagnetic, piezoelectric, and triboelectric nanogenerators, respectively. The optimal strategies and techniques to enhance these energy capacities are compiled in this paper.

**Graphical Abstract:**

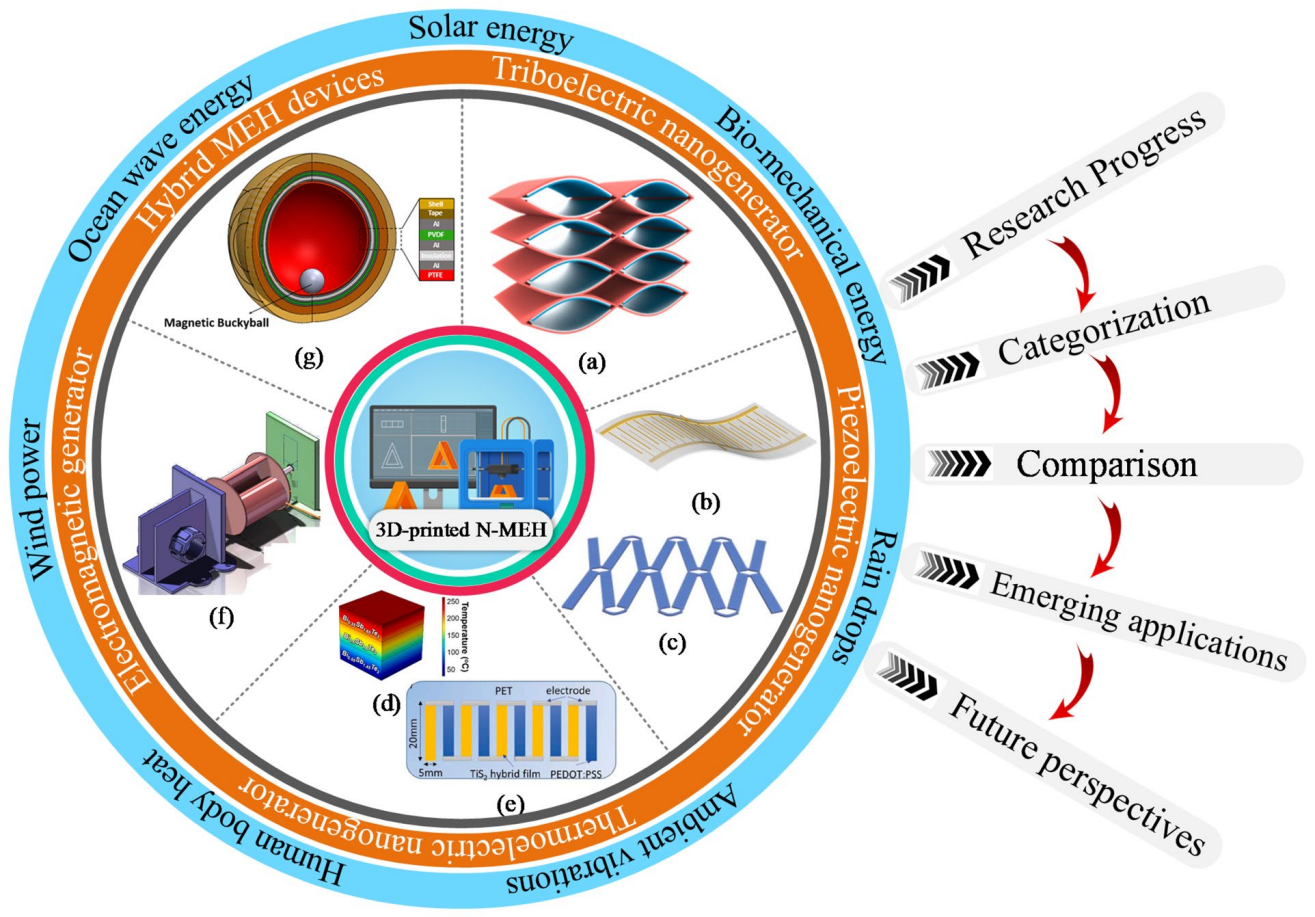

**Supplementary Information:**

The online version contains supplementary material available at 10.1186/s40580-021-00289-0.

## Highlights

- The current technology evolution and advancements in 3DP-NMEHs were evaluated.
- Categorization and comparison of 3DP-NMEHs were performed in terms of inputs and energy conversion.
- The emerging applications of 3DP-NMEHs were discussed briefly.
- The potential challenges confronted by 3DP-NMEHs and respective recommendations were summarized.
- Sustainable use of recyclable waste generated during the COVID-19 pandemic was proposed.

**Supplementary Information:**

The online version contains supplementary material available at 10.1186/s40580-021-00289-0.

## Introduction

In recent years, the robust advancement in the miniaturized and diversely functionalized microelectronic systems caused the development of smart nano-devices such as watches, health monitoring sensors, phones, and wearable gadgets. This has changed our lifestyle by enabling us to interact with others and confront disasters and diseases through self-powered nano-energy devices [1, 2]. Although the energy output of nano-devices is minimal compared to conventional energy powerplants and other resources [3, 4]. However, it has stimulated the development of self-powered gadgets and sensors for personal use with a wide range of applications. Harvesting energy using standalone nano-devices to provide clean and affordable energy to everyone is also a promising contribution to fulfilling Sustainable Development Goals (SDG 7) under the 2030 sustainability agenda implemented by United Nations [5, 6].

The growth of rapid manufacturing technologies has revolutionized the perception of nano-mechanical energy harvesting in the past decade [7]. Low-power nano-mechanical energy harvesting (3DP-NMEHs) systems [8] have been extensively employed for innovative electronic applications such as biomedical health monitoring sensors, pacemakers, watches, cell phones, self-powered wireless sensor nodes, self-powered water desalination, ocean navigation, surveillance, and structural monitoring [9].

Batteries are restricted by low energy densities, uncertain/limited lifetime, bulky size, hazardous chemicals, risks of overcharging, and expensive recycling/replacement/recharging; henceforth, various studies have been conducted to harvest energy directly from renewables [10], including wind [11], ocean waves [12], solar irradiations [13, 14], raindrops [15], biomechanical motions, bodily heat, sound [16] and railway vibrations [17] through various compact, cheap, and easily replaceable energy conversion technologies such as electromagnetic, piezoelectric, triboelectric, thermoelectric and pyroelectric nanogenerators [18], for powering wireless sensor networks [19] anywhere and anytime.

**Fig. 1:**
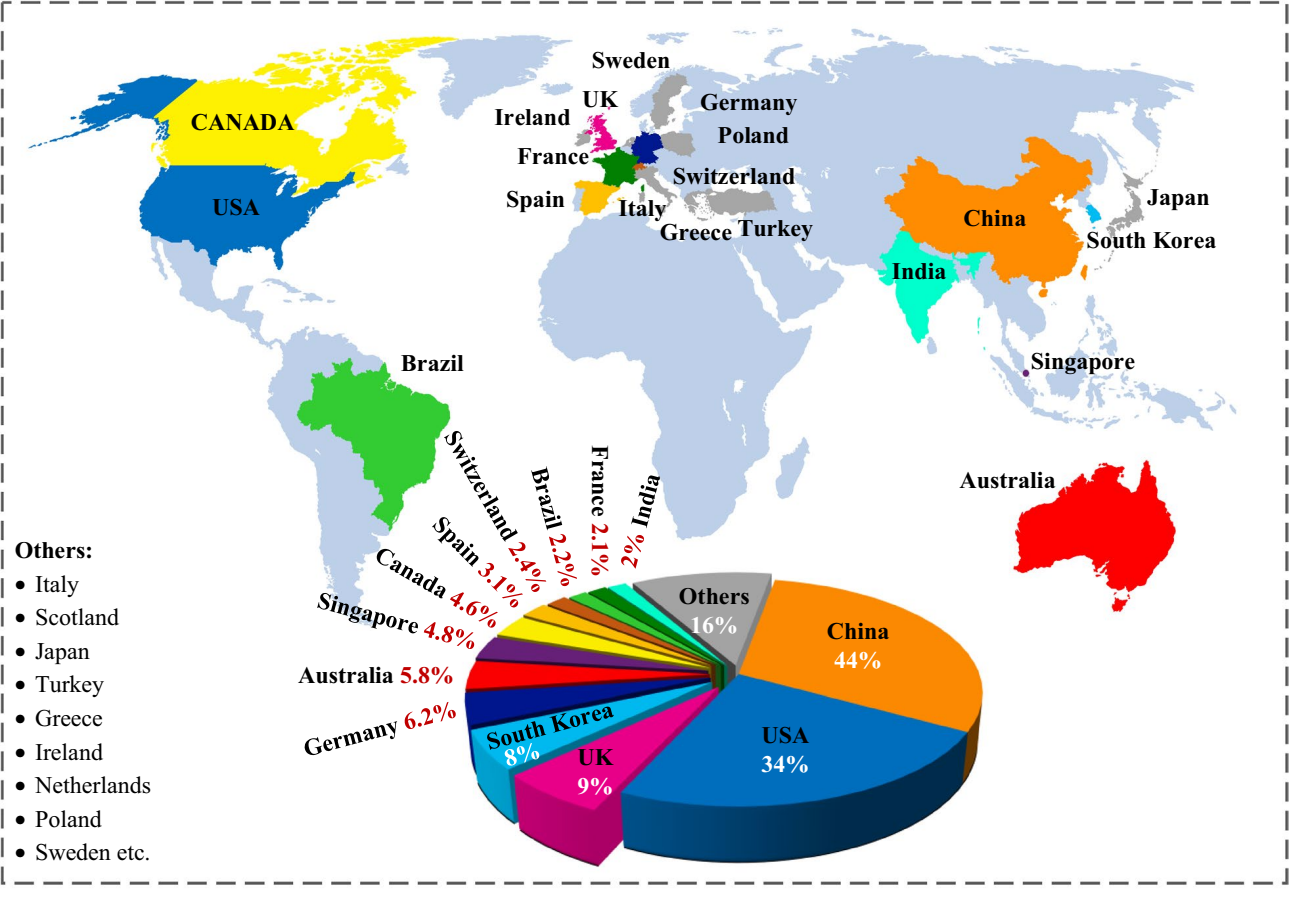
Contribution of the most prolific countries in the flourishment of 3D-printed mechanical energy harvesting systems

Additive manufacturing has advantages of accurate patterning, architectural customization, easy implementation, optimal mechanical resilience [20], lower environmental impact [21], sustainable/flexible production, faster speed, high-fabrication compatibility, less human intervention [22], relief from post-treatment, and minimal wastage/usage of materials [23, 24], over conventional manufacturing. With these benefits, 3D-printed structural frames [25], casings, shells, blades [26], substrates [27], piezoelectric [28], and triboelectric materials [29] have been widely utilized in nanogenerators. Moreover, it enables the use of biodegradable and recyclable materials for the fabrication of NMEH devices. Figure 1 shows the flourishment of 3D printed nano MEH mechanisms in various countries. China and the USA are on the top of the list with 44% and 34% contributions, respectively, holding more than half of the publications on the 3DP-NMEHs.

Tol et al. [30] developed a 3D-printed phononic crystal lens for elastic wave energy harvesting. The design parameters, including aperture size and gradient coefficient, were optimized numerically under plane wave excitation to achieve optimal performance. Lee et al. [27] demonstrated a 3D-printed hybrid coaxial TENG inspired by a crank engine to harvest large current from the ambient rotational motions at high frequencies. The prototype revealed an energy conversion efficiency of 17% at optimal output performance. Lu et al. [31] constructed a bidirectional gear transmission TENG using 3D printing to harvest energy from ambient vibrations to power a digital thermometer. The design parameters, including flywheel mass and the triboelectric film size, were optimized for optimal energy harvesting. The proposed design developed a short-circuit current of 14 μA, an open-circuit voltage of 450 V, and an output energy of 2.4 mJ.

Han et al. [26] developed a 3D printed miniature NMEH to harvest wind energy. The maximum output power from the device was 0.31 W at a 7% of maximum energy conversion efficiency. In another previous study, a 3D printed grating disk type NMEH device was presented as a power source for wireless electronic systems to harvest wind energy by Seol et al. [25]. The prototype developed a short-circuit current of 18.9 μA, an open-circuit voltage of 231 V, and maximum output power of 2.2 mW.

In a previous study, a ship-shaped hybridized nanogenerator was demonstrated for energy harvesting from water waves. The model produced a peak output power of 9 mW at a resistive load of 100 Ω, sufficient for powering self-powered positioning and seawater self-desalination. The experimental investigation demonstrated that the desalination rate could reach up to 30% in 180 min. Other demonstrated applications for the proposed MEH system include driving radio-frequency emitters for wireless positioning systems in ocean and rescue systems. Gao et al. demonstrated a 3D printed hybrid blue energy harvester based on a rotating gyro structure [32]. The proposed device was validated as a self-powered tracking system for an autonomous underwater vehicle in addition to an inertial sensor for marine equipment. Similarly, 3D printed NMEH systems, and solar energy trees have been developed for solar energy harvesting applications.

A 3D printed flexible triboelectric nanogenerator was demonstrated as a blue energy harvester and self-powered electro-Fenton degradation system for wastewater treatment by methylene blue degradation [29]. The peak density of output power, short circuit current, and an open-circuit voltage obtained from the prototype were 6 Wm^−2^, 2 mA, and 610 V, respectively. It was revealed that the methylene blue degradation efficiency could reach up to 98% within approximately an hour. Yuan et al. [33] developed a 3D-printed piezoelectric nanomechanical energy harvesting device for self-powered sensor applications. The PENG generated a maximum voltage of 74 V and power density of 478 μWcm^−2^. The potential applications for the proposed NMEH were self-powered artificial skin and tactile sensors. Zhou et al. proposed a 3D-printed stretchable PENG with a non-protruding kirigami structure for wearable electronic devices [34]. The prototype PENG could be stretched up to > 300% strain and was suggested for application as a self-powered gait sensor.

Yang et al. [35] constructed a thermoelectric generator (TEG) using multi-material 3D printing. The device could generate a peak output power density of 260 mWcm^−2^ and an efficiency of 9% at a temperature difference of 236 °C. A flexible thermoelectric device composed of a p-type and n-type organic superlattice film deposited onto the flexible PET substrate was developed for low-temperature applications [36]. The power factor of 210 μW m^−1^ K^−2^ was achievable at ambient room temperature. At ∆T = 70 K, a peak power density of approximately 2.6 Wm^−2^ was recorded for the proposed TEG. Han et al. [26] demonstrated a 3D printed miniature electromagnetic (EMG) NMEH driven by wind speed. The peak output power of 0.3 W at an energy conversion efficiency of 7% was achieved experimentally from the prototype. A 3D printed hybrid 3D activity inertial sensor was developed based on the combination of EMG, TENG, and PENG nanogenerators that could be used in inertial sensing in 6 different directions [37]. The structure consisted of magnetic buckyballs captured inside a 3D–printed spherical casing. The inner walls of the shell were deposited with layers of aluminum, PTFE, and PVDF films, whereas wire coils surrounded the outer surface. The sensor performed effectively in state monitoring of human activities and motion recognition applications. Moreover, the prototype could sense the x, y, and z components of the acceleration during linear motion and yaw, roll and pitch components of angular velocity during rotational motion.

Despite many advantages related to NMEHs, some challenges still need to be addressed. For example, efforts are required to employ advanced and simpler methods to modify the surface morphology of the materials for optimal nanopatterning to improve the surface work function and output power. Unfortunately, most printable polymers are unsuitable for implementation in biomedical implants, encapsulations, and wearable electronics. In addition to a few 3D printing methods currently used in developing NMEHs, demonstrating new optimal 3D-printing techniques to develop intelligent nanogenerator structures and materials is crucial. Furthermore, prototyping of integrated nanogenerators and functional accessories such as implants, sensors, and actuators is challenging [38]. Due to the unavailability of favourable processes and materials, fabrication of the coil spools and permanent magnets used in electromagnetic (EMG) generators is not currently achievable through rapid prototyping techniques. In addition, the requirement of bulky gear mechanisms for enhancing speed to maximize output power also hinders the assembly of compact EMGs [39]. Due to their high-temperature processing, it is complicated to 3D print rare-earth dielectric materials for piezoelectric nanogenerators (PENG) [40]. Triboelectric nanogenerators are highly compatible with rapid prototyping technology; however, it is restricted by the wear of the modified surface morphology caused by friction and adhesion of the printed nanopatterns [9]. In this regard, significant efforts are needed to develop optimal fabrication procedures and novel materials for fabricating nano-MEH devices.

A comprehensive study focusing on in-depth technology evolution, applications, problems, and future trends of specifically 3D printed nano-MEH systems with an energy point of view is rarely conducted. Therefore, this paper looks into the technologies, energy harvesting sources/methods, performance, implementations, emerging applications, potential challenges, and future perspectives of additively manufactured nano-mechanical energy harvesting (3DP-NMEH) systems. The prevailing challenges concerning renewable energy capacities, optimal energy scavenging, power management, material functionalization, sustainable manufacturing strategies, new materials, commercialization, and hybridization were highlighted. For sustainable energy generation and medicinal purposes, a strategy is proposed for sustainable applications of recyclable municipal and medical waste generated during the COVID-19 pandemic. Finally, recommendations for future research are presented concerning the significant issues hurdling the optimal exploitation of renewable energy resources through NMEHs.

## Nano-mechanical energy harvesting (NMEH)

**Fig. 2:**
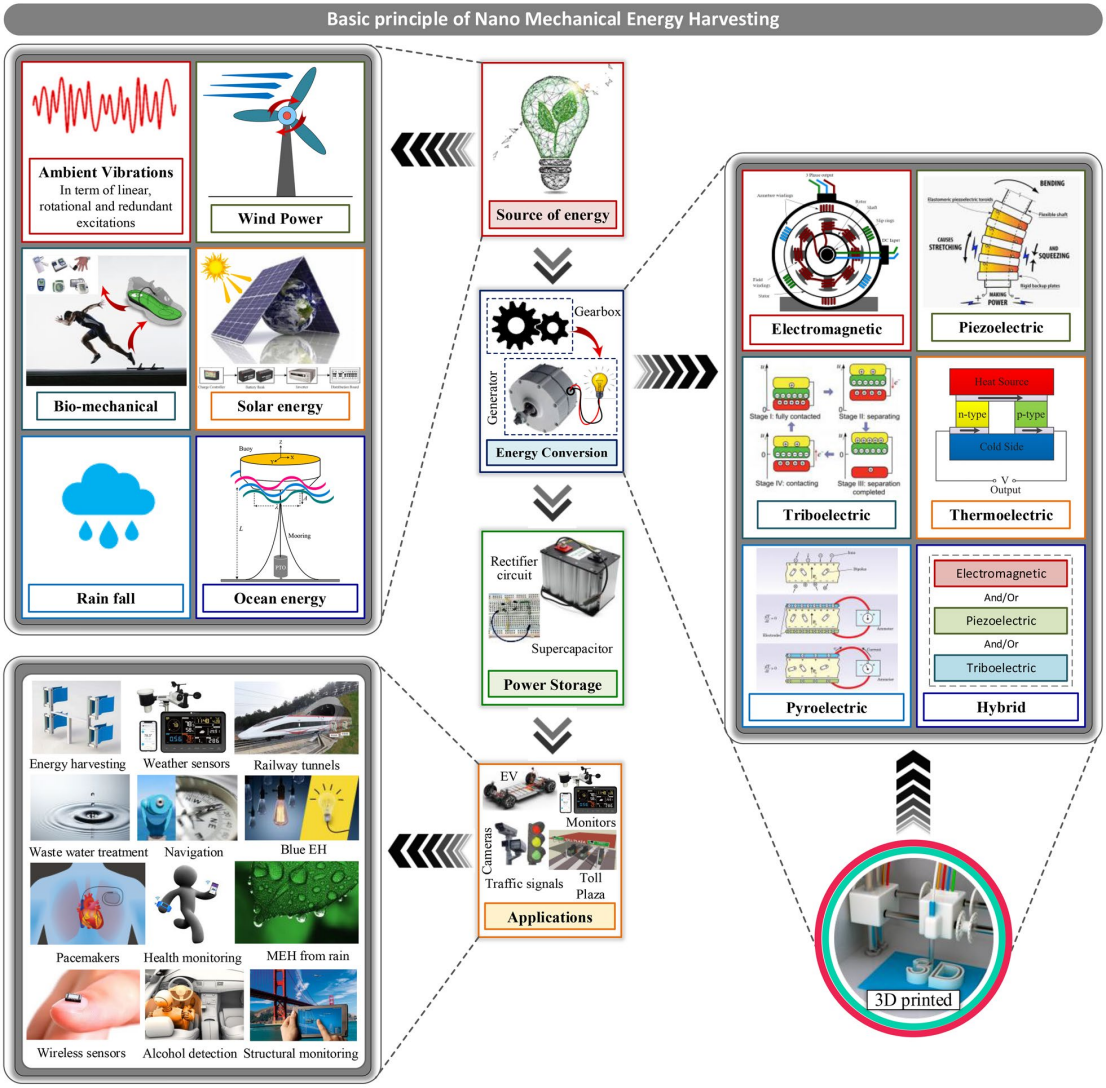
Schematic of nanomechanical energy harvesting along with various energy sources, energy conversion methods and potential applications

Harnessing energy from the ambient energy resources in the environment is an adequate substitute for the battery-based operation for low-power and self-powering devices. Besides, utilizing the harvested power near the energy source can eliminate the need for long transmission cables and power storage systems. Recently it has been revealed that micro/nano energy harvesting systems such as triboelectric, electromagnetic, piezoelectric, and electrostatic transducers can provide electrical power reaching from a few tens to hundreds of μW [8]. Figure 2 shows the layout of nano-mechanical energy harvesting with various ambient sources, 3DP-based manufactured energy conversion technologies, and potential applications reviewed in this study. The process consists of four modules.

I. Source of perturbation or ambient energy
II. Energy conversion mechanism
III. Post-processing and power storage
IV. Suitable applications

The most frequently used energy sources are wind, solar irradiation [41], ocean waves, human motion, raindrops [15], and ambient vibrations. The energy captured by these ambient sources can be transformed into useful form through several energy conversion phenomena such as electromagnetic, piezoelectric, triboelectric [42], thermoelectric, pyroelectric, and hybrid systems. Various studies have employed 3D printing for the development of structural components [25] of energy conversion devices such as turbine blades [26], casings, substrates [27], piezoelectric materials [28], solar energy trees, nano-structures for triboelectric nanogenerators, and many others. A few applications of NMEH are powering wearable devices, wireless sensor networks, navigation devices, wastewater treatment, charging mobile phones, biomedical healthcare systems, lighting LED, weather sensors, structural health monitoring, and cloud-based data transfer systems [8]. The output power can be post-processed before utilization, using complex rectifying and power management circuits. Batteries and supercapacitors are widely used to store harvested energy.

However, potential challenges still exist, such as the development of printing materials, printing of nanostructures, surface modifications, matching ambient vibration frequencies, scalability, usage location, energy conversion rate, and mass production. In addition, the significance of choosing a suitable battery, supercapacitor, or energy storage system must not be underrated. The impedance and capacity of the energy storage device must match the pulsed output of the NMEH device. Table 1 shows the distribution, significance, and performance of nano-mechanical energy harvesting systems over various sources of MEH and categorization with respect to output power. Table 2 elaborates the features and characterization of some previously developed nano-MEH systems to harvest renewable energy from natural resources such as wind, ocean, and solar energy.

**Table 1:** Distribution and significance of nano-mechanical energy harvesting systems over various renewable energy sources of MEH and categorization concerning output power

Source	Nano	References	Energy conversion	Input	Output	Efficiency
Road profile	✓	[43]	Piezoelectric EH structure	2–4 mm, 5–10 Hz	3 mW	–
	×	[44]	Chessboard sliding plate on the road	–	66 W	62.4%
	×	[45]	Piezoelectric MEH using road bump	10–30 km/h, 38–53 kg	4.1 W	13%
	×	[46]	High-efficiency MEH paver	80 kg	12 W	50%
	✓	[47]	MEH from multi-directional vibration	5 km/h	1.4 mW	–
Railway track vibrations	×	[48]	Electromagnetic energy convertor	1–2 Hz, 6 mm	6.5 V	56%
	✓	[49]	Thermoelectric EH using railway track	–	317 mW	60%
	✓	[50, 51]	Smart railway monitoring system	10–30 Hz	548 mW	–
	×	[52]	Vibration EH using multi-frequencies	5.6 Hz	1.5 W	–
	×	[53]	The electromagnetic based EH from the railroad	-	10–100 W	74%
Shock absorber vibrations	×	[54]	MEH from hydraulic shock absorber	5–25 mm, 1–5 Hz	397 W	50%
	×	[55]	Electromagnetic shock absorber EH for railway cars	2–4 mm, 2–4 Hz	1.2 W	68%
	×	[56, 57]	Regenerative mechanism for shock absorber	5–10 mm, 1–3 Hz	0.5 W	84%
	✓	[58]	Self-powered sensor nodes for freight rail transport	80 km/h	263 mW	65%
	×	[59]	Energy regenerative shock absorber based MEH	2.5–7.5 mm, 1–2.5 Hz,	4.3 W	55%
Ocean wave	×	[60]	Ocean-wave based energy harvesting mechanism	Wave height of 0.2 m	63 W	–
	×	[61]	The floating WEC	0.8 m, 0.76 Hz	–	39%
	×	[62]	WEC with power take-off mechanism	8 mm, 3 Hz	–	67%
	✓	[63]	Oscillating buoy as WEC	15 mm, 1.2 Hz	3 V	57%
	×	[54]	Hydraulic energy regeneration from shock absorbers	72 km/h	397 W	50%
Wind energy	✓	[64]	Piezoelectric EH from wind energy	6.5 m/s	25 mW	–
	✓	[65]	Wind EH using fibre composites	7.5 m/s	0.53 mW	–
	✓	[66]	High-performance piezoelectric EH for wind energy	2.1 m/s	1.2 mW	–
	✓	[67]	A rotational piezoelectric wind energy harvester	14 m/s	160 V, 2566 μW	–
	✓	[68]	Vibro-impact dielectric wind EH	4 m/s	0.72 mW	–
Bio-mechanical energy	✓	[69]	Scavenging energy from human motion	5 Hz	9 mW	8%
	✓	[70]	Electromagnetic EH from human movements	9 km/h	85 mW	92%
	✓	[71]	MEH for ultra-low frequency vibrations	4 Hz	5.3 mW	–
	×	[72]	A human motion-based vibration energy harvester	4.8 km/h	5.1 W	–
	✓	[73]	Biomechanical energy harvesting pavement	30 Hz	300 mW	–

**Table 2:** Features and characterization of some previously developed nano-systems to harvest renewable energy from natural resources (wind, ocean and solar resources)

Energy conversion	Mechanism	Input	Output	Advantages ($\uparrow$) and disadvantages (↓)
Electromagnetic generators (EMG)	Portable wind energy harvester based on S-rotor and H-rotor	5–12 m/s	108 mW, 23.2%	($\uparrow$) powers the monitoring sensors in railway tunnels ($\uparrow$) uses hybrid S-rotor and H-rotor
	Double-Skin Façade system for harvesting wind energy	3–8 m/s	1110 W/m^2^	($\uparrow$) low turbulence and uniform flow due to cavity (↑) provides a wide range of angles for incident wind
	Galloping, vortex shedding, flutter, and aerodynamic instability	2–6 m/s	1 W	(↑) based on wake galloping (↑) a simpler mechanism for structural health monitoring system (↑) powers wireless sensors
Piezoelectric nanogenerators (PENG)	The flutter of a flexible piezoelectric membrane	9 m/s	5 mW/cm^3^	(↑) simple inverted flag orientation (↑) Self-aligning capability (↑) can operate in low-speed regimes
	Vortex-induced vibration-based piezoelectric EH	1–1.4 m/s	0.6 mW	(↑) facilitates Y-shaped attachments on bluff body (↑) provides an enhanced energy harvesting efficiency
	MEH is composed of permanent magnets, rotor, piezoelectric stack, and flexure mechanism	100 rpm	0.2 mW	(↑) simple and compact design (↑) optimal performance with a larger power output
Pyroelectric (PEG)/ Thermoelectric generators (TEG)	Flexible vortex generator or turbulator	1–25 m/s	3 $\mu$ W/cm^2^	(↑) Flexible structure with un-interrupted energy output (↓) possesses low pyroelectric coefficient
	Harvesting solar and wind energies using thermal oscillations through sustainable PEG	2.5–5.3 m/s	421 $\mu$ W/cm^3^	(↑) provides high power density (↓) power density depends on the intensity of the solar irradiations and wind speed
Triboelectric nanogenerators (TENG)	A rotary TENG based on mechanical deformation of multiple plates	15 m/s	39 W/m^2^	(↑) facilitates the application of polymer nanowires (↑) can be used as a self-powered wind speed sensor
	TENG-based windmill composed of nanopillar-array architectured layers	14–15 m/s	568 V, 26 μA	(↑) simple and cheap fabrication (↑) high output and optimal performance (↑) high stability
	Pendulum-based TENG using a pendulum structure with high energy conversion efficiency	2 m/s, 2 Hz	56 V	(↑) superior durability (↑) ultrahigh sensitivity (↑) long-time operation (↑) energy harvesting from wave and wind

The references of the research papers cited in this table are provided in the Additional file 1

## Advancements in 3D printed Nano-MEH systems

**Fig. 3:**
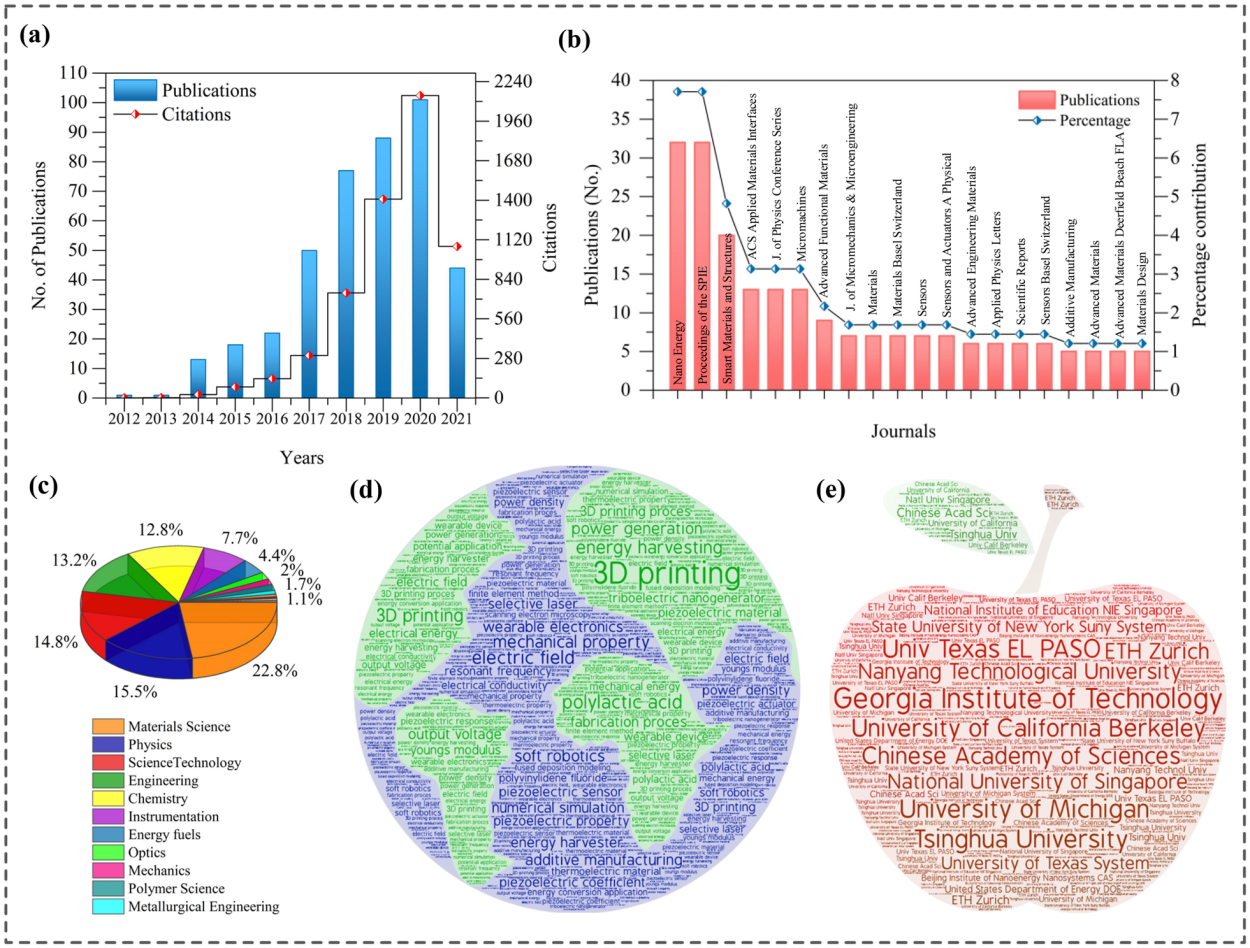
Research progress in 3D printed MEH systems **a** Growth in publications and citations related to 3D printed MEH devices, **b** leading journals in the flourishment of 3DP nano MEH research, **c** significant research areas of the published literature, **d** the most influential keywords in the study of 3D-printed nano MEH systems, **e** the most prolific institutions contributing to the enhancement of nano MEH research, Results were obtained from postprocessing of the data, retrieved from Web of Science using search keywords {TOPIC: ((“3D Printed” OR “3DP” OR “3D printing”)) AND TOPIC: ((“energy convertor” or “Triboelectric” OR “Piezoelectric” OR “Thermoelectric” or “nanogenerator” OR “Nano-energy harvester” OR “Biomechanical energy” OR “human movements” OR “Nano energy” or “Mechanical energy” OR “Energy harvesting” OR “linear generator” OR “Energy harvester” OR “Electromagnetic generator”)), Refined by: Document types: (Article or Review) and Research domains: (Science & Technology) and Languages: (English), Timespan: 2010–2021. Databases: WOS, CSCD, DIIDW, KJD, MEDLINE, RSCI, SCIELO}

Figure 3 signifies the technology evolution pathways of 3D printed mechanical energy harvesting systems in terms of bibliometric parameters such as publication growth, top contributing research areas, frequently investigated keywords, and the most prolific journals and institutions globally. The published literature, including original research articles, were obtained from Web of Science and investigated in CiteSpace software to visualize the specialties. It can be observed in Fig. 3a that the number of publications on 3DP-NMEHs was ignorable till 2013 and dramatically increased after 2013, reaching above a hundred in 2020. The number of citations drastically grew after 2016. Nano Energy, Proceedings of the SPIE, and Smart Materials & Structures were the top three most prolific journals contributing to the advancement of 3DP-NMEHs research, as shown in Fig. 3b. The highly dynamic research areas involved in the flourishment of the research are Materials Science, Physics, Science & Technology, Engineering, and Chemistry (Fig. 3c). The most frequently studied keywords related to the domain are 3D printing, energy harvesting, mechanical modulations, polylactic acid (PLA), triboelectric nanogenerator (TENG), and piezoelectric nanogenerators (PENGs) (Fig. 3d). The institutions publishing the most significant publications in the 3DP-NMEH research field are the Georgia Institute of Technology, University of California Berkeley, Nanyang Technological University, Chinese Academy of Sciences, and Univ Texas EL PASO, as shown in Fig. 3e.

### Potential areas of sustainable energy development using 3D printed devices

**Fig. 4:**
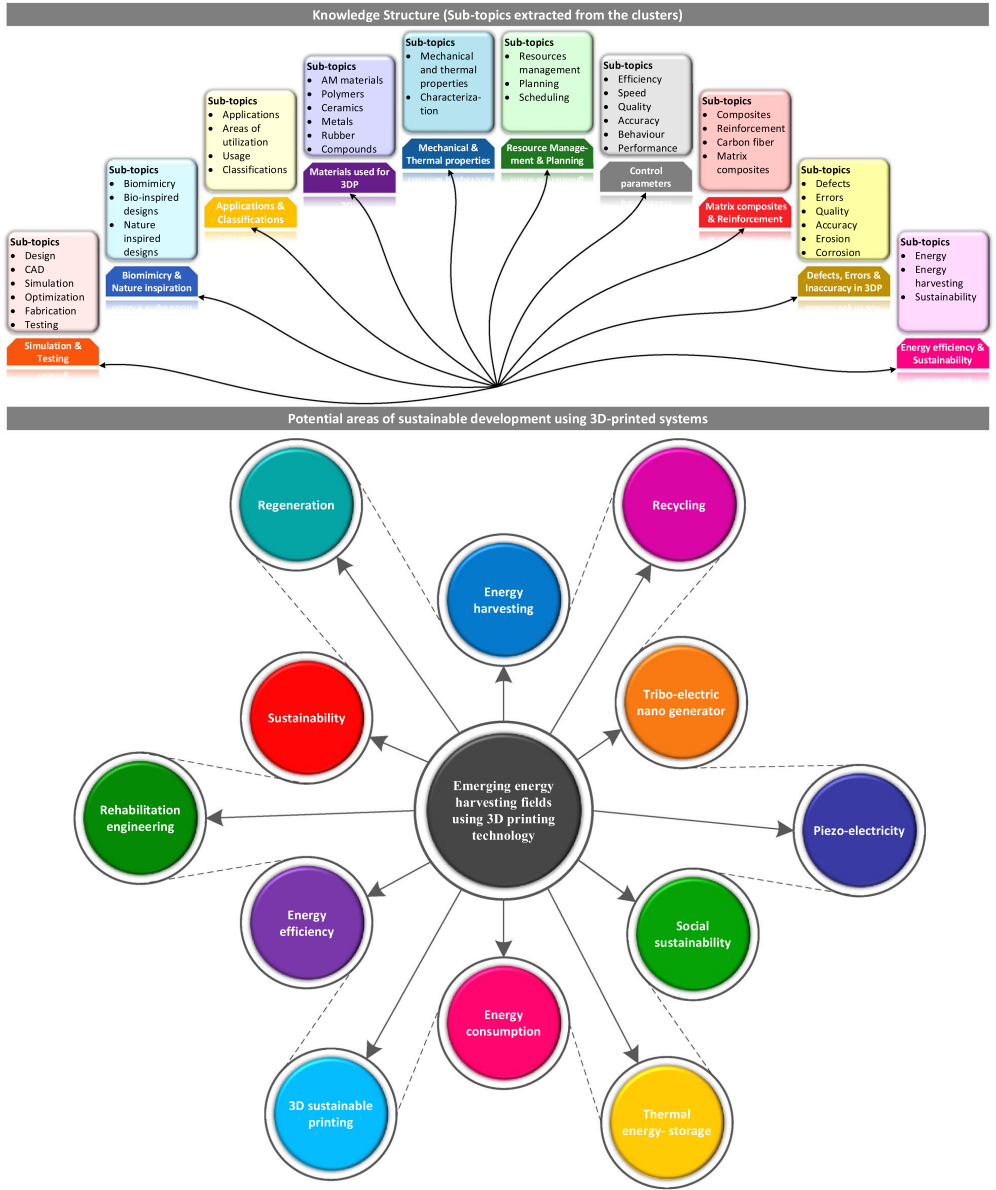
Energy harvesting as a prominent domain in 3D-printing research and significant areas of MEH using 3D-printed devices

To determine the inter-disciplinary research fields where 3D printed components are being extensively used, a simulation study was conducted using CiteSpace software based on the literature retrieved from Web of Science databases. A knowledge structure was developed after detailed analyses to evaluate the network of these research domains, as shown in Fig. 4. It can be noticed that “sustainable energy” and “energy harvesting” are among the top ten research fields related to 3D printing. It shows the emerging significance of 3D printing in developing mechanical energy harvesting (MEH) mechanisms. In addition, the emerging fields of MEH using 3D printed components are mapped in Fig. 4. Numerous devices have been reported to be fabricated using the integration of 3D printed parts to harvest energy from various sources such as human activities [31] and joint movements, wind [26], ocean waves, sound [16], rain droplets, and other ambient vibrational energy sources [30]. The wind and ocean energy harvesting devices have been commonly fabricated using 3D printed substrates, structural frames, conductive electrodes [74], blades [75], rotors, printed electronics [76, 77], shells, and casings. Additive manufacturing facilitates the utilization of biodegradable and reusable materials for the manufacturing of nano-MEH systems. Moreover, the portability of the small and compact 3D-printed mechanisms has inspired the concept of nano-energy. However, the application of 3D printed parts in the development of medium to large-scale mechanical energy harvesting systems has been rarely reported. In future MEH research, significant efforts are required to optimize the printing materials and printing techniques to develop durable, reliable, renewable, and mechanically and thermally strong functional components for large power-extracting (greater than 1 Watt) MEH systems such as blue EH, wind turbines, regenerative shock absorbers, wave energy converters and railway-vibrations based MEHs.

## Resources and methods of 3D-printed Nano-MEH systems

### Renewable energy sources

**Fig. 5:**
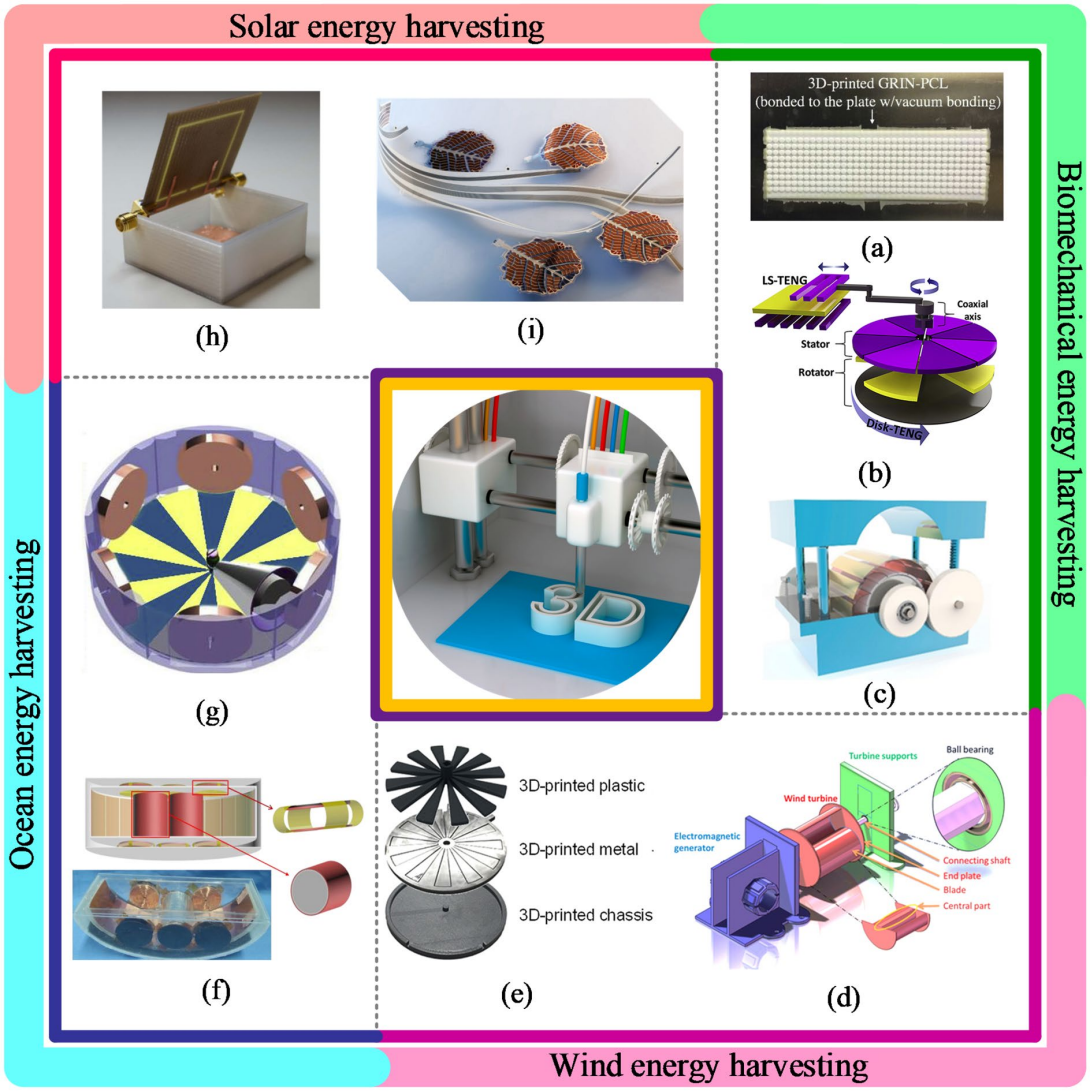
Various sources of MEH through 3D-printed MEH devices **a** 3D-printed phononic crystal lens for elastic wave energy harvesting [30] **b** 3D-printed hybrid coaxial TENG inspired by a crank engine [27] **c** Bidirectional gear transmission triboelectric nanogenerator fabricated using 3D printing [31] **d** 3D printed miniature electromagnetic energy harvester driven by airflow [26] **e** All 3D printed grating disk type TENG [25] **f** ship-shaped hybridized nanogenerator [78] **g** triboelectric-electromagnetic rotating gyro structured blue energy harvester [32], **h** Solar energy harvester with a 3-D printed package [84] **i** 3D printed solar energy trees (Image credits: alternative-energy-news.info/) (Images are re-used with the permission of the publisher)

The 3D printed nanomechanical energy harvesting systems can be categorized based on energy sources and energy conversion methods. Figure 5 shows significant energy sources used to harvest mechanical energy, such as ocean waves, wind, solar energy, biomechanical triggering, and ambient vibrations in the environment. Tol et al. [30] developed a 3D-printed phononic crystal lens for elastic wave energy harvesting, as shown in Fig. 5a. The design parameters, including aperture size and gradient coefficient, were optimized numerically under plane wave excitation to achieve optimal performance. Lee et al. [27] demonstrated a 3D-printed hybrid coaxial TENG inspired by a crank engine to harvest large current from the ambient rotational motions at high frequencies, as illustrated in Fig. 5b. The prototype revealed an energy conversion efficiency of 17% at optimal output performance. Lu et al. [31] constructed a bidirectional gear transmission TENG using 3D printing to harvest energy from ambient vibrations to power a digital thermometer, as shown in Fig. 5c. The design parameters, including flywheel mass and the triboelectric film size, were optimized for optimal energy harvesting. The proposed design developed a short-circuit current of 14 μA, an open-circuit voltage of 450 V, and an output energy of 2.4 mJ.

Han et al. [26] developed a 3D printed miniature NMEH to harvest wind energy, as shown in Fig. 5d. The maximum output power from the device was 0.31 W at a 7% of maximum energy conversion efficiency. A 3D printed grating disk type NMEH device was presented as a power source for wireless electronic systems in a previous investigation to harvest wind energy by Seol et al. [25], as given in Fig. 5e. The prototype developed a short-circuit current of 18.9 μA, an open-circuit voltage of 231 V, and maximum output power of 2.2 mW.

A ship-shaped hybridized nanogenerator was developed in a previous study for energy harvesting from water waves [78], as shown in Fig. 5f. The model produced a peak output power of 9 mW at a resistive load of 100 Ω, sufficient for powering self-powered positioning and seawater self-desalination. The experimental investigation demonstrated that the desalination rate could reach up to 30% in 180 min. Other demonstrated applications for the proposed MEH system include driving radio-frequency emitters for wireless positioning systems in ocean and rescue systems. Gao et al. demonstrated a 3D printed hybrid blue energy harvester based on a rotating gyro structure [32], as shown in Fig. 5g. The proposed device was validated as a self-powered tracking system for an autonomous underwater vehicle in addition to an inertial sensor for marine equipment.

Organic solar cells have limited applications due to low energy conversion efficiencies compared to their inorganic counterpart. It is due to the short-range light absorption, low charge mobility, and electrical conductivity of the prevailing organic materials. Carbon nanomaterials, including graphene and carbon nanotubes, are characterized by high mobility, electrical conductivity, and unique optical characteristics, making them suitable for organic solar cell applications [79]. Moreover, graphene-based energy storage devices have also been employed for power management of these nano-devices [80]. Recently developed colloidal quantum dots (CQDs) have revolutionized photovoltaics, and the resultant devices are capable of cheaper solution processes and a tunable bandgap [81]. Organic and metallic monovalent cations-based perovskite solar cells are newly developed solar devices that are less toxic than lead-based solar cells [82]. The new 3D printed NMEH systems and solar energy trees [83] developed for solar energy harvesting applications are shown in Fig. 5h, i.

### Energy harvesting methods

**Fig. 6:**
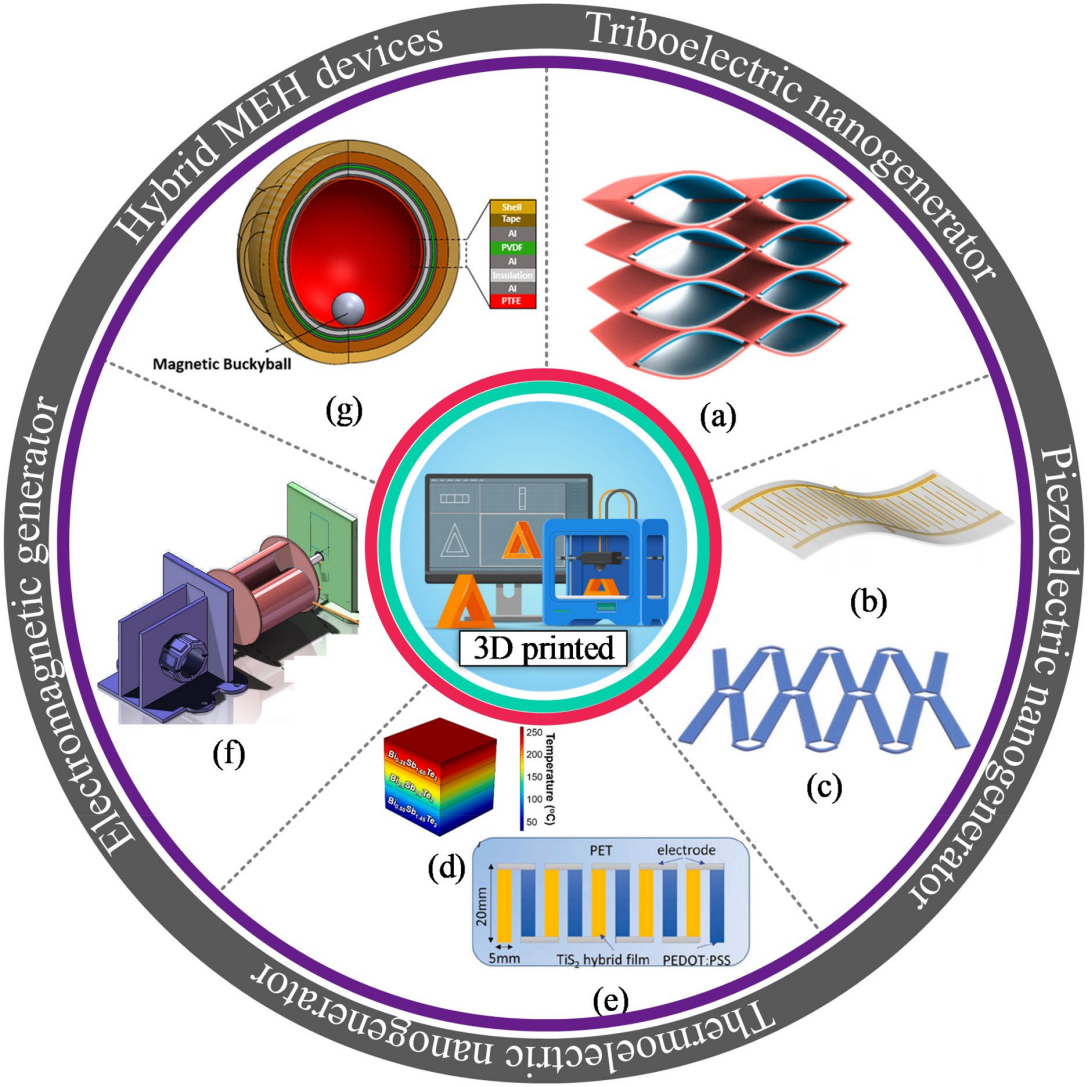
Classification of 3D printed energy harvesting devices based on working principle **a** 3D printed flexible triboelectric nanogenerator as a self-powered electro-Fenton degradation system [29] **b** 3D-printed piezoelectric device for self-powered sensor application [33] **c** 3D-printed stretchable piezoelectric nanogenerator with non-protruding kirigami structure [34] **d** 3D printed BiSbTe thermoelectric generator [35] **e** Thermoelectric device composed of a p-type and n-type organic superlattice film deposited onto the flexible PET substrate [36] **f** 3D printed miniature electromagnetic energy harvester driven by airflow [26] **g** 3D printed hybrid 3D activity inertial sensor based on EMG, TENG and PENG nanogenerators [37] (Images are re-used with the permission of the publisher)

The energy conversion methods primarily utilized for 3DP-NMEH systems include electromagnetic, piezoelectric, triboelectric, thermoelectric, and hybrid combinations, as shown in Fig. 6. A 3D printed flexible triboelectric nanogenerator was demonstrated as a blue energy harvester and self-powered electro-Fenton degradation system for wastewater treatment by methylene blue degradation [29], as shown in Fig. 6a. The peak density of output power, short circuit current, and an open-circuit voltage obtained from the prototype were 6 Wm^−2^, 2 mA, and 610 V, respectively. It was revealed that the methylene blue degradation efficiency could increase up to 98% within approximately an hour.

Yuan et al. [33] developed a 3D-printed piezoelectric nanomechanical energy harvesting device for self-powered sensor applications, as shown in Fig. 6b. The PENG generated a maximum voltage of 74 V and power density of 478 μWcm^−2^. The potential applications for the proposed NMEH were self-powered artificial skin and tactile sensors. Zhou et al. proposed a 3D-printed stretchable PENG with a non-protruding kirigami structure for wearable electronic devices [34], as shown in Fig. 6c. The prototype PENG could be stretched up to > 300% strain and was suggested for application as a self-powered gait sensor.

Yang et al. [35] constructed a thermoelectric generator (TEG) by multi-material 3D printing, as shown in Fig. 6d. The device could generate a peak output power density of 260 mWcm^−2^ and an efficiency of 9% at a temperature difference of 236 °C. A flexible thermoelectric device composed of a p-type and n-type organic superlattice film deposited onto the flexible PET substrate was developed for low-temperature applications [36], as shown in Fig. 6e. The power factor of 210 μW m^−1^ K^−2^ was achievable at ambient room temperature. At ∆T = 70 K, a peak power density of approximately 2.6 Wm^−2^ was recorded for the proposed TEG.

Han et al. [26] demonstrated a 3D printed miniature electromagnetic (EMG)-based NMEH driven by wind speed, as shown in Fig. 6f. The peak output power of 0.3 W at an energy conversion efficiency of 7% was achieved experimentally from the prototype. Figure 6g highlights a 3D printed hybrid 3D activity inertial sensor based on the combination of EMG, TENG, and PENG nanogenerators [37]. The structure consisted of magnetic buckyballs captured inside a 3D–printed spherical casing. The inner walls of the shell were deposited with layers of aluminum, PTFE, and PVDF films, whereas wire coils surrounded the outer surface. The sensor performed effectively in state monitoring of human activities and motion recognition applications. Moreover, the prototype could sense the x, y, and z components of the acceleration during linear motion and yaw, roll, and pitch components of angular velocity during rotational motion.

#### Electromagnetic energy harvesters

**Fig. 7:**
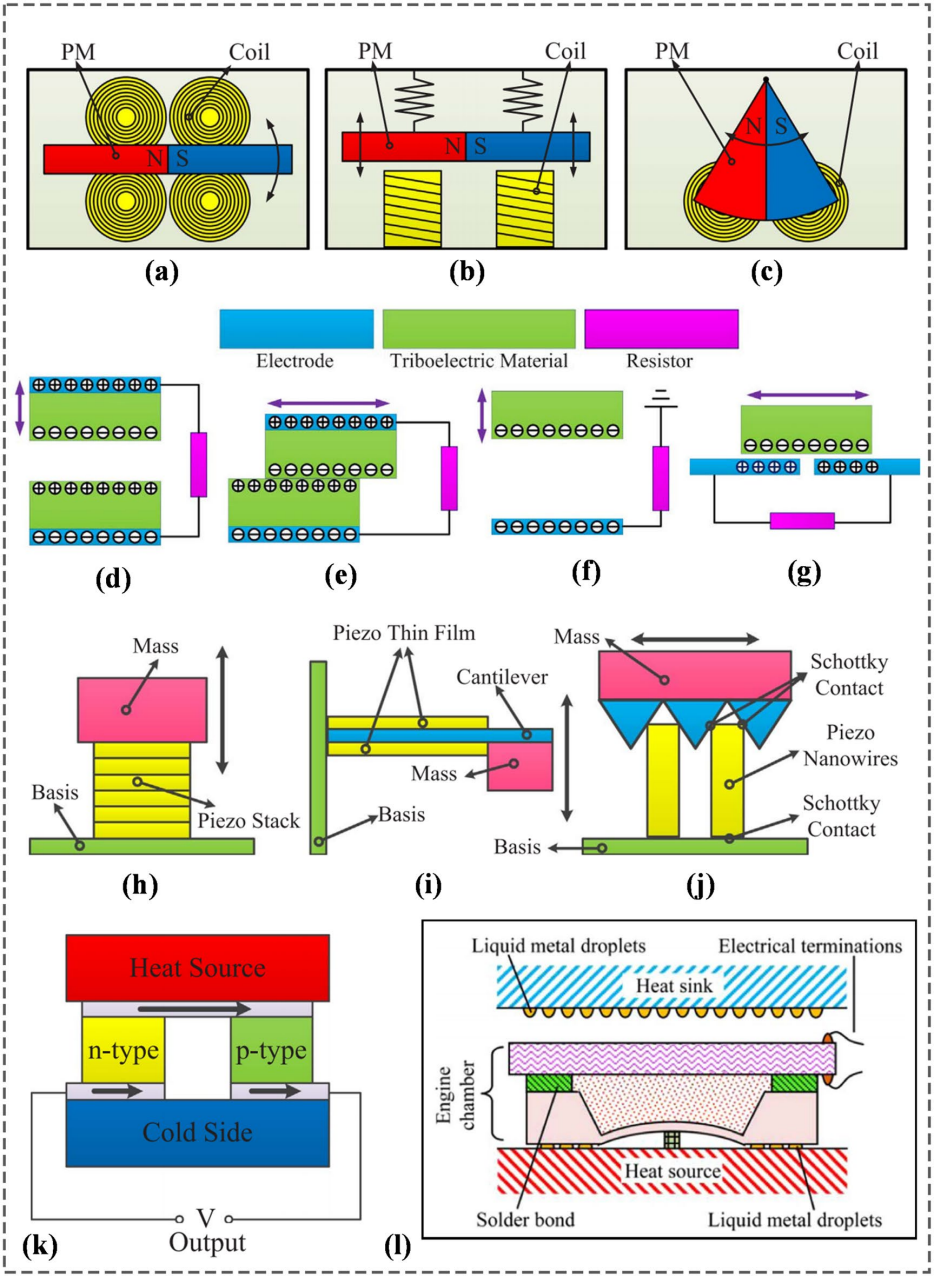
Working modes of electromagnetic energy harvesters: **a** rotational, **b** oscillatory and **c** hybrid; working modes of triboelectric nanogenerators **d** vertical contact-separation mode, **e** in-plane contact-sliding mode, **f** single-electrode mode and **g** freestanding mode working modes of piezoelectric energy harvesters: **h** d_33_ working mode, **i** d_31_ working mode and **j** piezotronic working mode; **k** schematic diagram of a thermoelectric nanogenerator **l** schematic of the micro pyroelectric energy harvester [39] (Images are re-used with the permission of the publisher)

Electromagnetic generator (EMG) based NMEH devices work on the principle of Faraday’s law of electromagnetic induction, a relationship between the induced electromotive force across the coil and time rate of change of the magnetic flux through the coil. The relative motion between the rotor coils and surrounding magnets induces a current in the external circuit. The coils and magnets can be fixed or movable. In Fig. 7a–c, three different working layouts of EMGs are illustrated [39] based on the types of mechanical motions involved.

- Rotation-based EMGs—are miniaturized electromagnetic generators that convert the continuous rotational motions of the magnets or armature into electrical output, as shown in Fig. 7a.
- Oscillatory EMGs—are based on the oscillatory motions between the magnets and coils for energy conversion, as shown in Fig. 7b.
- Hybrid EMGs—utilize an imbalanced structure to harvest energy from chaotic or random vibrations by converting rectilinear motion into rotational motion, as shown in Fig. 7c.

**Fig. 8:**
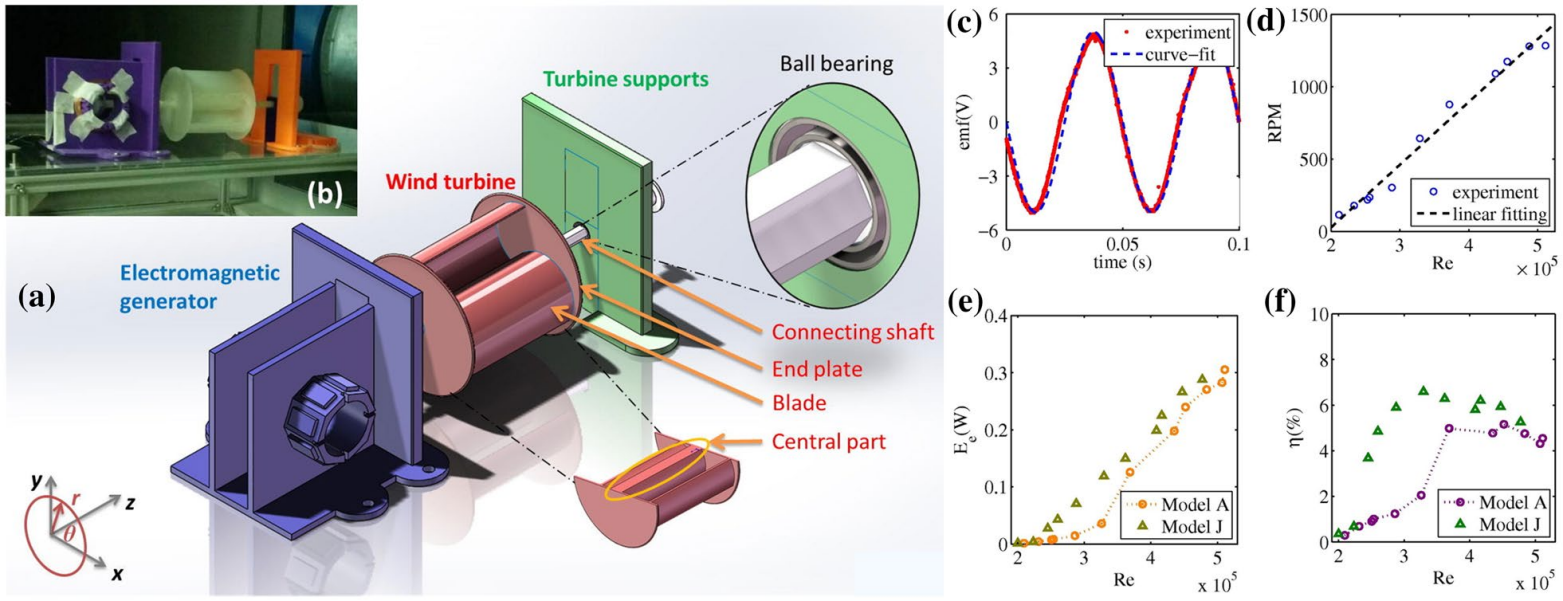
**a** CAD model of the air-driven 3D printed EMG energy harvester, **b** prototype for the experimental testing in the wind tunnel **c** output voltage with time **d** RPMs with Reynolds number (Re) **e** output power with Re **f** energy conversion efficiency with Re [26] (Images are re-used with the permission of the publisher)

3D printing of conventional EMG-based MEH devices is challenging due to the unavailability of favourable printing processes and materials. For example, 3D printing of the coil spools and permanent magnets used in electromagnetic generators is not achievable currently. In addition, several factors such as low output voltage, high output current, unavoidable coil losses, and the requirement of bulky gear mechanisms for enhancing speed to maximize output power levels add to the difficulty of 3DP-based development of micro/nanoscale EMG MEH devices. Many researchers have conducted efforts to develop miniature structures for EMG-based MEHs. Han et al. [26] developed a 3D printed miniature electromagnetic energy harvester driven by airflow, as shown in Fig. 8a, b. The maximum output power from the device was 0.31 W at a 7% of maximum energy conversion efficiency, as shown in Fig. 8c–f. The rotational speed of the turbine and output power was increased with increasing Reynolds number (Re). However, the optimal energy conversion efficiency was restricted by friction with the increase in the rotating speed of the turbine. The proposed 3D printed EMG-based wind-driven MEH system was demonstrated as an efficient strategy to harvest renewable wind energy and can be used to supply power to HVAC ventilation systems and household electronic appliances. Gao et al. demonstrated a 3D printed EMG blue energy harvester based on a rotating gyro structure [32]. The proposed device was validated as a self-powered tracking system for an autonomous underwater vehicle in addition to an inertial sensor for marine equipment.

#### Triboelectric nanogenerators

Triboelectric nanogenerator (TENG), working on the principle of electrostatic induction and contact electrification, has excellent potential in nanoscale energy harvesting applications. Owing to the high energy conversion efficiency, low fabrication cost, and optimal output power density, triboelectric nanogenerators are widely used for MEH from wind [85], water waves [86], human motion [87, 88], and ambient vibrations [89]. Various techniques have been utilized, such as spin coating [90], etching [91], and electrospinning, to improve the frictional effect of the triboelectric materials. This is accomplished by developing the micro or nanostructures on the surface to enhance the effective frictional contact areas between the TENG surfaces. 3D printing facilitates high material-usage efficiency to fabricate objects from complex CAD models in a shorter period. Therefore, it has been widely employed to manufacture casings, blades, shells, tubular components, structural frames, nano-patterns, surface modifications, and friction materials. TENG holds exceptional compatibility with 3D printing technology for the following reasons:

- Most of the materials used for the development of TENGs are compatible with 3D printing. For example, the structural supports are made of plastics, the triboelectric materials are mostly polymers, and the electrodes are conductors, and all of these can be fabricated through 3DP.
- The manufacturing requirements of TENGs are compatible with the 3D printing process as there is no need for vacuum apparatus or high-temperature treatment after fabrication. Moreover, the device performance is not compromised by 3DP.
- 3DP facilitates accurate and simple structural control, which is advantageous for TENG fabrication. For instance, the dampers and springs can easily be 3D printed, and interdigitated configurations can be 2D printed, improving EH performance.
- 3D printing offers customization in design considering the optimization of various complicated structural parameters

Due to frictional contact between two materials with different electronegativities, opposite charges accumulate at the contact surfaces and remain for a long time even after the separation of the surfaces. If the triboelectric materials are integrated with electrodes connected to an external circuit, the cyclic separation and contact of triboelectric surfaces develop a charge on the surfaces, causing an alternating electric current in the circuit. It is due to the electrostatic induction and varying equivalent capacitance between the surfaces. In Fig. 7d–g, four different working modes of TENGs [92] are shown as described below,

- Vertical contact-separation mode—is based on the cyclic contact and separation between the two horizontal triboelectric surfaces [93], as shown in Fig. 7d.
- In-plane sliding mode—is based on the rotational or sliding motions between the two planar contacting surfaces. The change in the out-of-contact area causes the generation of AC output in the external circuit [94], as shown in Fig. 7e,
- Single electrode mode—consists of a moving object (acting as an electrode) and the other electrode connected to the ground [95], as shown in Fig. 7f. Due to electrostatic screening, the current induced across the electrode is not very effective [96]; however, it has found wide applications due to the mobility of the triboelectric layer.
- Free-standing mode—eliminates electrostatic screening and utilizes the movement of a single triboelectric layer relative to the other two under-placed externally connected electrodes [97], as shown in Fig. 7g.

**Fig. 9:**
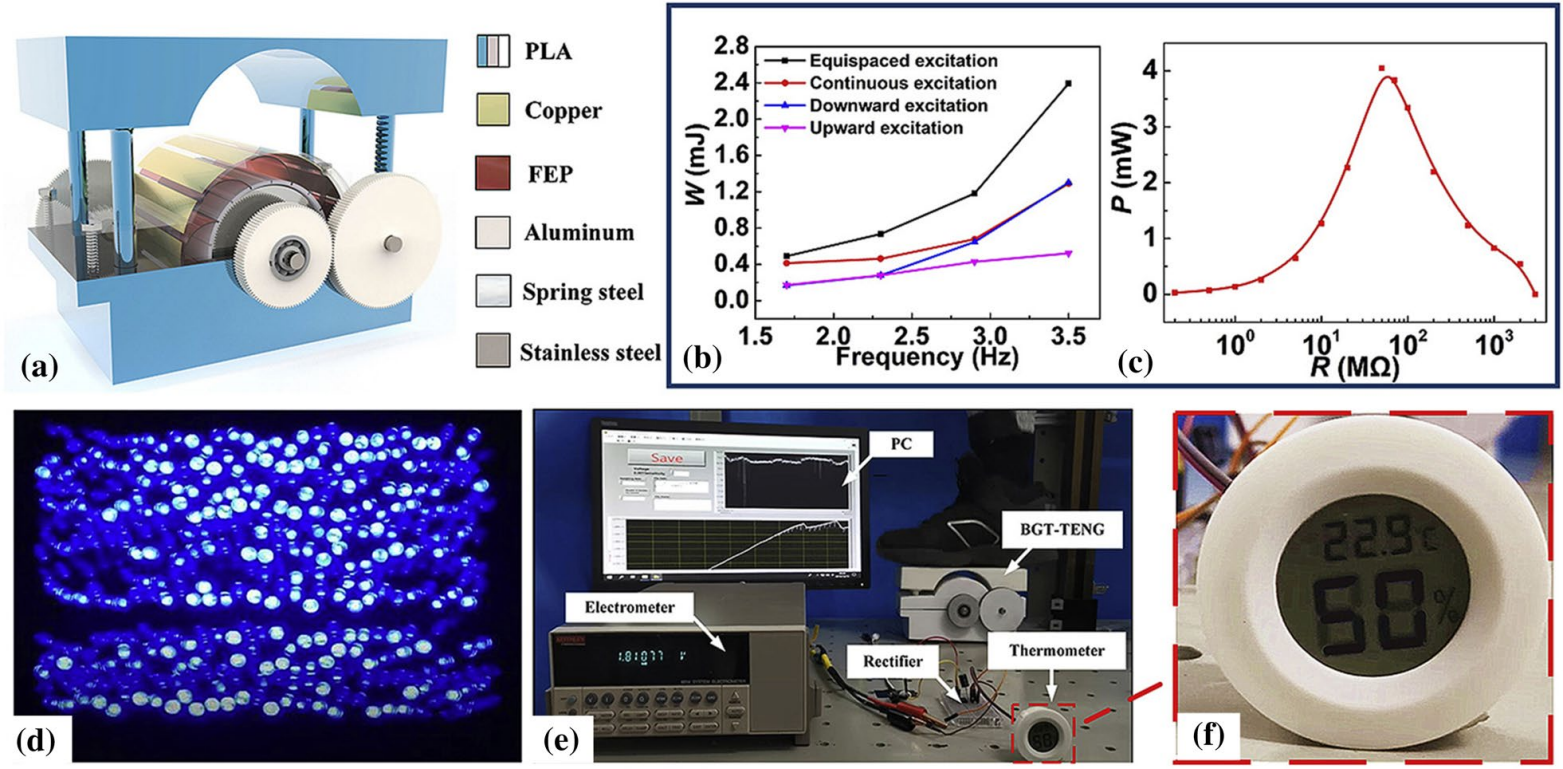
**a** 3D printing assisted bi-directional gear transmission TENG **b** output energy **c** instantaneous power output with varying external resistances **d** powering 375 LEDs **e** experimental setup **f** output power of the TENG used to run a thermometer [31] (Images are re-used with the permission of the publisher)

Lu et al. [31] demonstrated a bi-directional gear-transmission mechanism for TENG that could harvest mechanical energy during an entire stroke of excitation. The proposed design achieved 2.4 mJ with short-circuit current $I_{t}$ and open-circuit voltage $V_{t}$ of 14 μA and 450 V. Both $V_{t}$ and $I_{t}$ were observed to be increased with increasing the length of the triboelectric film but were limited to a certain magnitude due to resulting contact of the films with two adjacent metallic electrodes at larger film lengths. Maximum output energy was obtained at an optimal film length of 45 mm, and film installation angle of 30°. Figure 9a shows the proposed design of the 3DP-TENG, and the output energy and power trends are shown in Fig. 9b, c. The 3D printed TENG device was demonstrated to power 375 LEDs and a commercial thermometer, as shown in Fig. 9d, f. The testing facility used to evaluate the performance of TENG is shown in Fig. 9e. A linear motor (J-5718HBS401, Yisheng, China) was used to excite the TENG, and the output performance of the TENG was analyzed with a programmable electrometer (6514, Keithley, USA) integrated with a data acquisition module (PCI-6259, National Instruments, USA).

In contrast, LabVIEW software was employed to record the output electrical signals. Significant efforts in developing TENG-based 3D printed NMEH systems are summarized in Table 3 regarding working conditions, materials, output characteristics, and energy applications. Some common applications of 3D printed TENG nano-devices include wearable electronics, self-powered healthcare monitoring sensors, thermometers, sustainable removal of methylene blue (MB) emissions, sensors for subway tunnels, wireless sensor nodes, thermo-hygrometers, smartwatches, temperature/vibration sensors, biomechanical applications, driver habits-monitoring, and road condition analysis.

**Table 3:** Triboelectric nanogenerator based 3D printed energy harvesting devices, their output energy capacities and applications

Energy harvesting devices	Source of excitation	Excitations	Materials	Output	Applications
Wrist-wearable TENG device	Human wrist-motions	≤ 5 Hz	ABS, PLA	0.118 mW/cm^3^	Self-powered healthcare monitoring sensors
Bidirectional gear transmission based TENG	The motion of the human foot	3.5 Hz	PLA	4 mW	Thermometers
Elastic TENG based self-powered electro-Fenton system	Reciprocation by hand	2–5 Hz	Acrylic	1.95 W/m^2^	Sustainable removal of methylene blue (MB) emissions
Hybrid coaxial TENG	Rotary motion	100–400 rpm	ABS, acrylic	846.4 $\upmu$ W	Small toys
Wind-driven hybrid TENG nanogenerator	Slow speed wind	6 m/s	PLA	245 mW	Subway tunnel
Freestanding kinetic-impact-based TENG	Human motions	5 Hz	PLA	102.29 mW	Thermo-hygrometers, smartwatches
Flexible TENG for vibration energy harvesting	Vibrations	6 Hz	Acrylic	608.5 mW/m^2^	Portable and wearable sensors
3D-printed silicone-Cu fiber-based TENG	Human motion	≤ 5 Hz	Si elastomer	31.39 mW/m^2^	Biomechanical applications
Integrated flywheel & spiral spring TENG	Human foot motion	≤ 5 Hz	PLA	38.4 mJ	LEDs
Low-frequency resonant TENG nanogenerator	Manual vibrations	18 Hz	ABS	2.61 mW	Vibration sensors, recharging batteries
Novel sweep-type TENG	Rotary motion	1.2 m/s	PLA	400 V, 15 μA	Driver habits-monitoring
Mechanical frequency regulator based TENG	Human and windmill	10–50 Hz	PLA	17 V, 6.5 mA	Wireless node sensors
Water droplet vibrations based TENGs	Vibrations	1–30 Hz	ITO glass	7.55 μW	Self-powered electronic systems
Origami-tessellation-based TENG	Ambient excitations	3–16 Hz	Nylon	26.16 μW	Energy harvesting on road pavement
Galloping TENG based on two flexible beams	Wind energy	1.4–6 m/s	ABS, PET	200 V, 7 μA	Outdoor electric devices
Direction-switchable TENG	Human joint motions	5–15 cm/s	PLA	5 V, 10 μA	Temperature sensors
Rotary cam-based TENG	Rotary motion	300–1000 rpm	PLA	3.5 mW	Commercial and industrial applications
Nanopillar-array architectured TENG	Wind energy	14–15 m/s	PLA	568 V, 25.6 μA	Wind energy harvesting

The references of the research papers cited in this table are provided in the Additional file 1

*EMG* electromagnetic generator, *TENG* triboelectric nanogenerator, *ABS* acrylonitrile poly-butadiene styrene, *PLA* polylactic acid, *ITO* Indium tin oxide

#### Piezoelectric nanogenerators

Piezoelectricity describes the reversible interaction between electrical and mechanical states in specific materials. It refers to the development of electric potential across output terminals under applied mechanical stress [98]. The inverse piezoelectric effect is the indication of mechanical strain due to the applied electric field. Based on the working principle, PENGs can be categorized into three types: (1) the $d_{33}$ mode, (2) the $d_{31}$ mode, and (3) piezotronic mode, as shown in Fig. 7h–j. In $d_{33}$ mode, as shown in Fig. 7h, piezoelectric stacks are composed of various thin films connected mechanically in serial and electrically in parallel with each other. The piezoelectric layers are oriented in a specific manner to achieve an optimal voltage under the minimal strain (0.1%) for piezoelectric materials. Finally, the layers are integrated to make different stacks which effectively enhance the amount of accumulated charge. The piezoelectric stacks are placed on cantilever supporting structures in $d_{31}$ working mode, as shown in Fig. 7i. Various structures, including cymbals, bimorphs, and unimorphs, have been investigated as effective piezoelectric transducer structures. In the piezotronic layout, the Schottky barrier is provided between the electrodes and nanowires to control the electric flow, as shown in Fig. 7j.

**Fig. 10:**
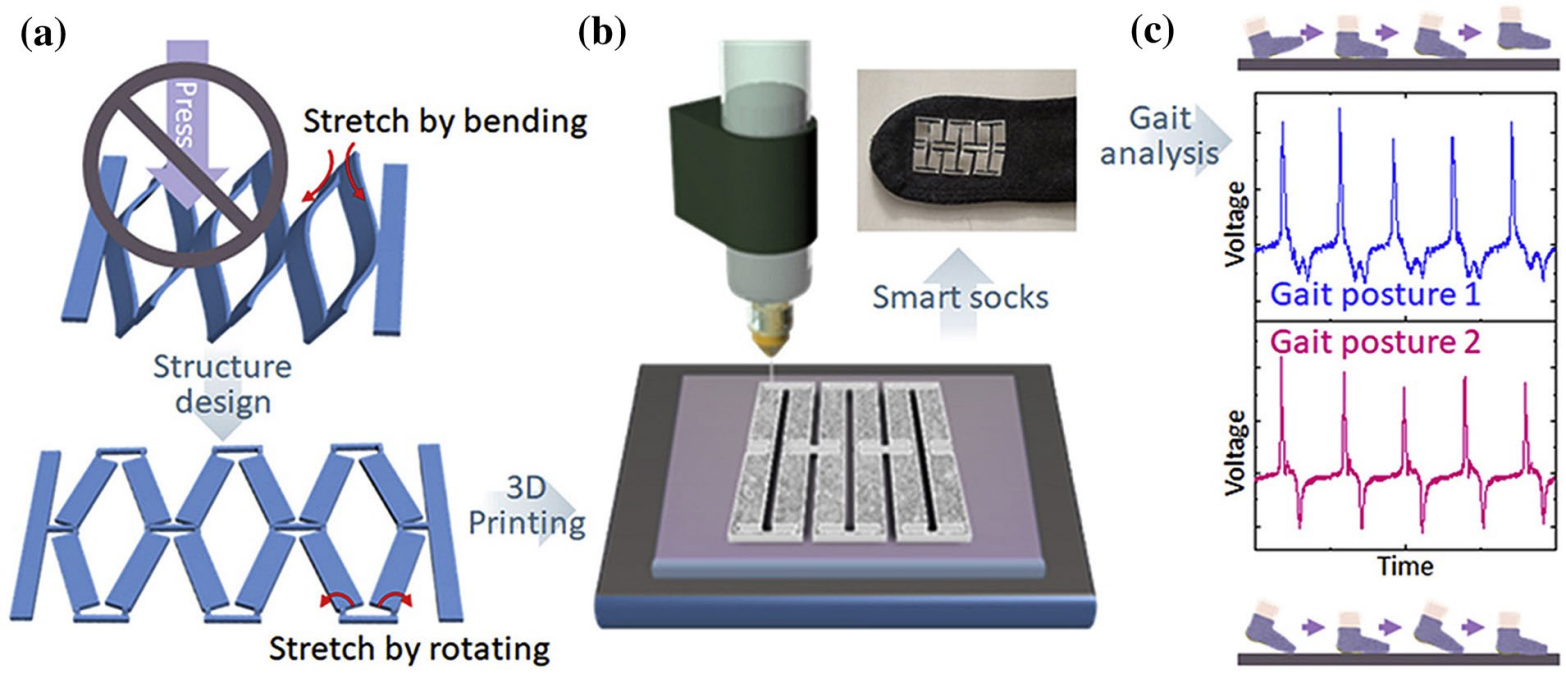
**a** 3D printed PENG with the kirigami structure **b** fabrication of 3D-printed PENG **c** output voltage of the printed PENG-integrated socks under different foot-stamping postures [34] (Images are re-used with the permission of the publisher)

Zhou et al. [34] developed an all 3D printed piezoelectric nanogenerator (PENG) using a non-protruding kirigami-like structure as shown in Fig. 10a. Figure 10b describes the 3D printing of the PENG, and Fig. 10c demonstrates the mounting of the PENG device on a sock with adhesive. It was observed that in normal stamping (casual walking), there is only one (> 0) peak and two (< 0) peaks. Whereas, when the toes are fixed on the ground, and only the heel is stamped, then one (> 0) peak and one (< 0) peak is obtained. Experimental results proved that the 3D printed PENG could detect various gait postures and pace frequencies, which could be a promising solution for detecting steppage gait and slap gait caused by some neurological ailments. Park et al. [99] developed stretchable nanocomposite-based PENG to harvest energy from the minute biomechanical motions inside the human body for self-powered wearable electronics and sensitive piezoelectric sensors. Significant contributions to piezoelectric-based 3D printed nano energy harvesting systems are highlighted in Table 4 regarding working conditions, materials, output characteristics, and applications. Some typical materials used in 3D printing of PENGs are photocurable resins, PVDF-TrFE [33], piezoelectric inks [100], and acrylic [37]. The typical applications of PENGS include energy focusing, ultrasonic sensing, self-powered conformal sensors, haptic sensing of a robotic hand, external stress stimulation, self-powered tactile sensors, artificial skin, gait sensors, body motion sensor, multi-axis rotation and acceleration inertial sensing, telemedicine applications, anthropomorphic grippers, flexible electronics, and force sensor applications.

**Table 4:** Piezoelectric nanogenerator based 3D printed energy harvesting devices, their output energy capacities and applications

Energy harvesting devices	Source of excitation	Excitations	Materials	Output	Applications
Piezoelectric ceramics for MEH	Vibrations	–	Photocurable resin	0.301 V	Energy focusing, ultrasonic sensing
Piezoelectric BNNTs nanocomposites	Biomechanical energy	10 Hz	Photocurable resin	24 mV/kPa	Conformal sensors, haptic sensing of robotic hand
3D-printed PVDF-TrFE piezoelectric film	Finger and wrist joints	0.5–4 Hz	PVDF-TrFE	73.5 V	External stress stimulation, tactile sensors, artificial skin
Stretchable kirigami piezoelectric nanogenerator	Vibrations from magnetic shaker	5 Hz	Piezoelectric ink	1.4 μW/cm^2^	Self-powered gait sensor
Stretchable piezoelectric nanogenerator	Vibrations from magnetic shaker	5 Hz	3D printable ink	0.29 V	Body motion sensor
3DAIS	3D vibration, rotation & human motion	2.5 Hz	Acrylic	0.19 µW	Multi-axis rotation and acceleration inertial sensing, telemedicine applications
Stiffness-tunable soft robotic gripper	Finger bending	1 mm/s	FLX9760, RGD8530	3 V	Anthropomorphic grippers
Ceramic-polymer composite	Universal testing machine	100 Hz	Grid-composite	270 mV	Flexible electronics, force sensors

The references of the research papers cited in this table are provided in the Additional file 1

*BNNTs* Boron nitride nanotubes, *PVDF-TrFE* Poly (vinylidene fluoride-co-trifluoroethylene), *3DAIS* 3D activity inertial sensor

#### Energy harvesting from heat

Energy harvesting from human body heat depends on the temperature change of the body and involves two types of mechanisms: the thermoelectric generators (TEGs) and the pyroelectric generators (PEGs). TEG is developed on the principle of thermoelectric conversion and converts the temperature difference across the device into an electrical signal [101], whereas PEG relies on the temporal temperature variations of the device. TEG is a thermocouple made of two different conductors in which two junctions are maintained at high temperature $T_{h}$ and low temperature $T_{c}$, respectively. As a result, an open-circuit voltage $V_{oc}$ directly proportional to the temperature difference ∆T is developed across the output terminals. Generally, a TEG is composed of many thermocouples comprising p-type and n-type doped elements [102]. The thermoelectric elements are integrated so that they are thermally connected in parallel and electrically connected in series. The basic schematic of a TEG is shown in Fig. 7k.

The pyroelectric generator (PEG) generates pyroelectric current due to a change in polarization orientation due to temperature fluctuations [103]. The basic schematic of a PEG is illustrated in Fig. 7l. A PEG based on a liquid–gas phase-change fluid and a temperature-dependent operational frequency [104] was demonstrated to achieve an output power of 40 mW at a ∆T of 80 K, as shown in Fig. 7l.

**Fig. 11:**
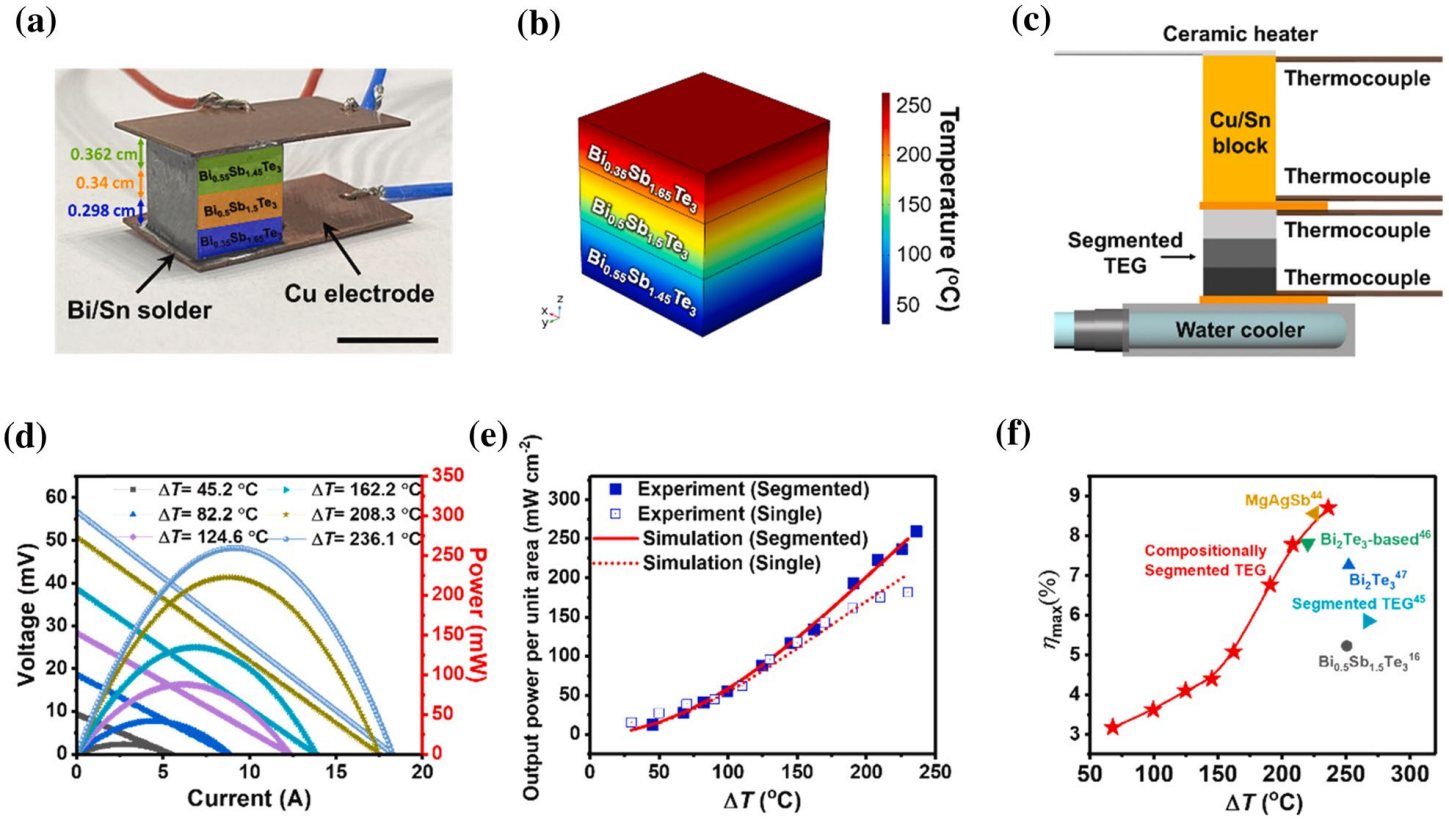
**a** A 3D printed compositional three-block BiSbTe thermoelectric generator **b** temperature gradient along the TEG layers evaluated by simulations **c** testing schematic to experimentally measure the output power and energy conversion efficiency of TEG **d** the output power and voltage of the proposed TEG at varying temperature difference **e** comparison of the simulated and experimentally measured power densities of the TEG as a function of the applied temperature difference **f** trend of energy conversion efficiency with ΔT [35] (Images are re-used with the permission of the publisher)

Yang et al. [35] designed a novel TEG using a multi-material 3D printing method and composition-persuaded BiSbTe material. The TEG could achieve a peak power density of approximately 259 mW/cm^2^ and an energy conversion efficiency of approx. 9% at a temperature difference of 236 °C. Figure 11a shows that a TEG with the p-type leg was manufactured by integrating the copper electrodes with the legs with Bi/Sn solder. Figure 11b shows the temperature distribution along the chipped TEG from the numerical study. The test scheme for experimental measurement of ΔT across the Sn/Cu block is shown in Fig. 11c. With an increase in the temperature difference, the voltage was increased to be maximum at 57 mV, as shown in Fig. 11d. The power density also directly relates with ΔT, and a peak power density of 259.3 mW/cm^2^ was observed experimentally (Fig. 11e). From Fig. 11f, it can be noticed that the maximum efficiency of almost 9% was achieved at the maximum temperature difference (236 °C). It was demonstrated that the efficiency obtained from the proposed TEG was higher than the previously reported TEGs. Some significant contributions in developing thermoelectric and pyroelectric-based 3D printed nano energy harvesting systems are presented in Table 5, along with working conditions, printable materials, output characteristics, and applications. The common applications of heat energy harvesting are wearable electronics, self-powered sensors, and microelectronic applications.

**Table 5:** Thermoelectric generator based 3D printed energy harvesting devices, their output energy capacities and applications

Energy harvesting devices	Source of excitation	$\Delta T$	Materials	Performance	Applications
Flexible thermoelectric power generator	Electric heater	30 K	TE materials	80 mV	Wearable electronics
Segmented thermoelectric generators	Ceramic heater	236 °C	BiSbTe-based viscoelastic inks	8.7%	Self-powered sensors
Conformal cylindrical thermoelectric generators	Hot water flowing through alumina pipe	39 °C	Bi_2_Te_3_-based inks	1.62 mW	–
3D printed SnSe thermoelectric generators	Thermoelectric tester	772 K	Tin selenide (SnSe)	20 µW	Solar cell applications
A flexible and stretchable organic thermoelectric device	Heating controller	75 K	Polyurethane/CNT nanocomposites	19.8 ± 0.2 µV/K	–
Shape-controllable thermoelectric devices	Heating rod	54.6 K	Bi_2_Te_3_/(PVP) composites	0.68 mW	–
Self-healing and stretchable 3D-printed TE device	Body temperature	7 K	PEDOT: PSS	12.2 nW	Flexible electronics
Thick printed TE generator	Microelectronic heat sink	40 °C	Bi_2_Te_3_-based TE ingot	10 W/cm^2^	Microelectronic applications

The references of the research papers cited in this table are provided in the Additional file 1

*SnSe* Tin selenide, *CNT* carbon nanotubes, *PVP* polyvinylpyrrolidone, *TE* thermoelectric, *PEDOT: PSS* poly(3,4-ethylenedioxythiophene) doped with polystyrene sulfonate

#### Hybrid 3DP-NMEH mechanisms

Various energy conversion systems such as EMG, TENG, PENG can be integrated for maximum energy harvesting from various ambient energy sources, such as wind, solar energy, ocean waves, body heat, and biomechanical energy. Usually, PENGs and TENGs are hybridized with other energy harvesters due to their structural multiplicity and flexibility. PENG-EMG and TENG-EMG hybrids are the most popular combinations for nano/micro-scale self-powering applications. Various hybrid energy harvesting systems have been reported to be fabricated through 3D printed structures, parts, substrates, blades, frames, shells, and casings [26, 78, 105].

**Fig. 12:**
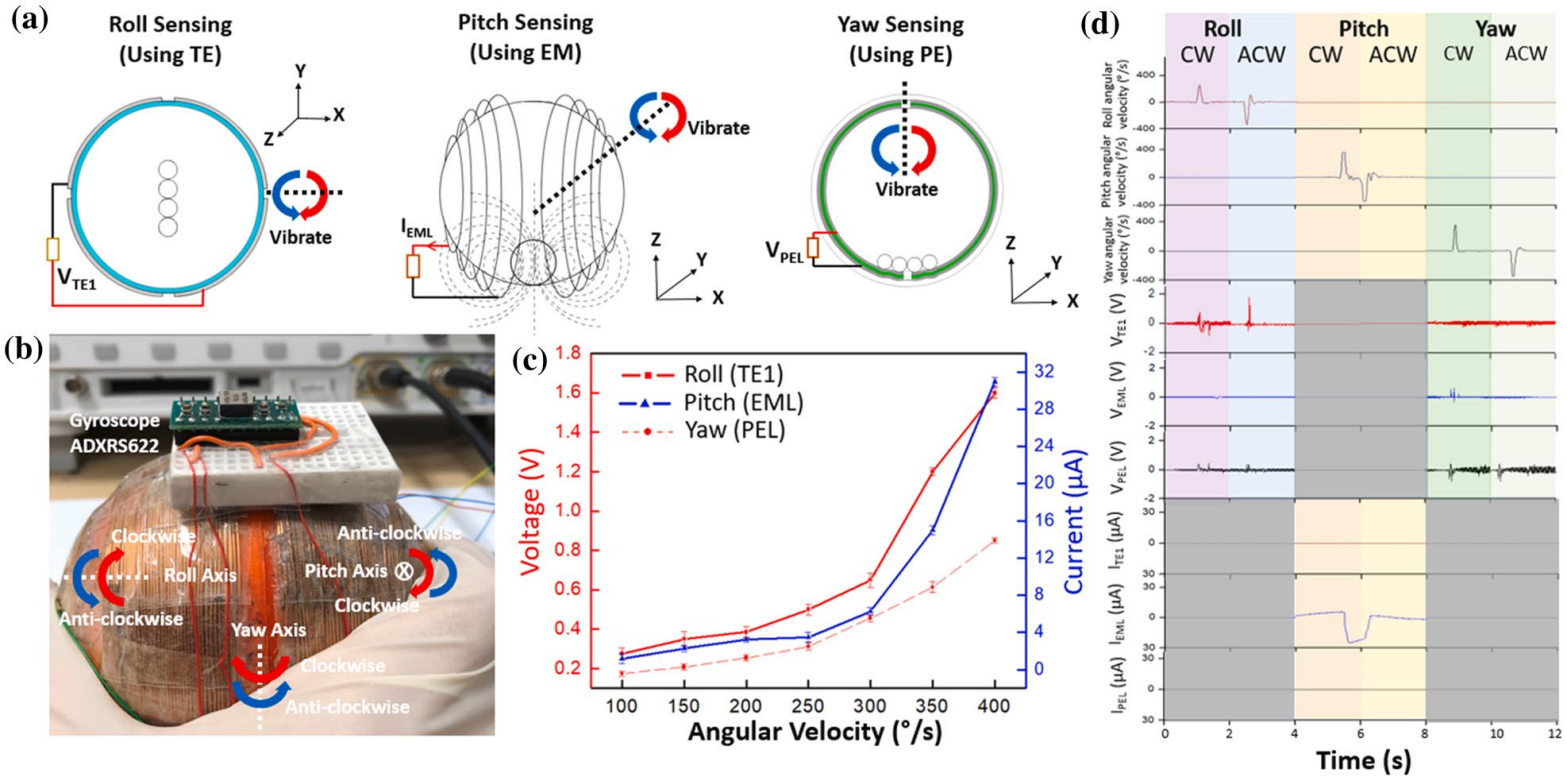
**a** Illustration of the application of TENG voltage in roll-sensing, EMG current in pitch-sensing and PENG voltage in yaw-sensing **b** prototype of the 3DAIS integrated with gyroscope **c** output current and voltage variation with angular velocity in yaw, roll, and pitch motions **d** profiles of angular velocities and output voltage from the hybrid EMG-TENG-PENG sensor for anticlockwise and clockwise roll, yaw and pitch movements, [37] (Images are re-used with the permission of the publisher)

Koh et al. [37] demonstrated the applications of a 3D printed hybrid EMG-TENG-PENG nanodevice in multi-axis acceleration/rotation inertial sensing and telemedicine. A spherical and symmetrical self-powered 3-dimensional sensor was proposed to record and measure the inertial movements in six directions. Figure 12a illustrates the application of TENG voltage in roll-sensing, EMG current in pitch-sensing, and PENG voltage in yaw-sensing. The prototype of the mechanism integrated with a gyroscope is shown in Fig. 12b. The variation of output current and voltage with angular velocity in yaw, roll, and pitch motions is plotted in Fig. 12c whereas, Fig. 12d shows the profiles of angular velocities and output voltage from the hybrid EMG-TENG-PENG sensor for anticlockwise and clockwise roll, yaw and pitch movements. The hybrid NMEH system could harvest energy using hybrid energy-conversion modules and be integrated into different human body parts for healthcare monitoring applications. Some significant contributions in developing hybrid 3D printed nano energy harvesting systems in terms of test conditions, printed materials, output characteristics, and applications are accessible in Table 6. Some recently reported common applications of hybrid nano-devices include wearable electronics, healthcare monitoring sensors, multi-axis acceleration & rotation inertial sensing, telemedicine applications, Internet-of-Things, HVAC (heating, ventilating, and air conditions) ventilation exhaust systems, self-functional tracking system, seawater self-desalination, and self-powered positioning.

**Table 6:** TENG, PENG, TEG and EMG based 3D printed hybrid MEH devices, their output energy capacities and applications

Energy harvesting devices	Source of excitation	Excitations	Materials	Output	Applications
Hybrid EMG-TENG wrist-wearable device	Human wrist-motions	5 Hz	ABS, PLA	0.118 mW/cm^3^	Wearable healthcare monitoring equipment
Hybrid EMG-TENG-PENG 3DAIS device	3D vibration, rotation & human motion	2.5 Hz	Acrylic	0.19 µW	Inertial sensing
Hybrid EMG-TENG wind-driven nanogenerator	Slow speed wind	6 m/s	PLA	245 mW	Subway tunnel monitoring sensors
Hybrid EMG-TENG device resonating at low frequency	Manual vibrations	18 Hz	ABS	2.61 mW	Vibration study
Hybrid TENG-EMG-PENG energy harvester	Hybrid step-servo motor	45 rpm (0.75 Hz)	ABS	712 μW, 31 mW, 6.4 μW	–
Solar & electromagnetic Energy harvesting System	Solar irradiance	100 mW/cm^2^	PLA	93 mW	Internet-of-Things
3D printed miniature EMG device driven by airflow	Wind energy, wind tunnel	–	ABS	0.305 W	HVAC (heating, ventilating, and air conditions) ventilation exhaust systems
Hybrid EMG-TENG rotating gyro structured blue EH	Blue energy	1.2 to 2.3 Hz	White resin	14.9 mW (EMG) 4.1 μW (TENG)	Self-powered and self-functional tracking system
Ship-shaped hybridized nanogenerator (SHNG)	Blue energy (linear motor)	2 Hz	PLA	800 µW (TENG) 9 mW (EMG)	Seawater self-desalination and self-powered positioning

The references of the research papers cited in this table are provided in the Additional file 1

*EMG* electromagnetic generator, *TENG* triboelectric nanogenerator, *PENG* piezoelectric nanogenerator, *3DAIS* 3D activity inertial sensor, *ABS* acrylonitrile poly-butadiene styrene, *PLA* polylactic acid, *EH* energy harvester

### Comparative assessment

A comprehensive comparative analysis was performed regarding advantages, disadvantages, strategies for effective energy harvesting, optimal locations for biomechanical energy harvesting, range of power output on nano-scale, and potential challenges of EMG, TENG, and PENG devices, as given in Table 7.

**Table 7:** Comparison of nanomechanical energy harvesting methods in terms of pros and cons, performance, techniques for efficient utilization, and challenges

Comparison/ types	Electromagnetic	Piezoelectric	Triboelectric
Pros	No requirement of contacts [106] No requirement of voltage source [106] Smaller mechanical damping [106] Higher current [107] Operation is durable and robust [107] Lower impedance [108]	Smaller mechanical damping [106] No need for a voltage source [106] Higher capacitance [107] No requirement of mechanical stoppers [106] High energy density [106] High output voltage (2–10 V) [106]	Flexibility in device structure [109] Higher power density [110] Can operate at lower frequencies [109] Easy to fabricate with nanoscale size [109] High energy conversion efficiencies [110]
Cons	Low efficiency at low frequency [107] Difficult miniaturization [111] High coil losses [112] Lower efficiency [112] Complex integration [106] Lower output voltage [106]	Low current and high impedance [108] Incompatible for CMOS process [111] Poor coupling at microscale [106] Difficult to integrate [106] Requirement of special piezoelectric materials [112] Can be self-discharged at lower frequencies [111]	Durability is not good [110] The mechanism is not fully understood [110] High voltage and low current [109] Challenging to be integrated [110] Electrostatic charge accumulation
Strategies for effective energy harvesting	Frequency up-conversion [113] Sprung eccentric rotor [114] Elimination of spring [115] Spring clockwork mechanism [116] Induce non-linearity [117]	Induce non-linearity [118] Proper circuit management [119] Frequency up-conversion [120] Use a double pendulum system [121]	Development of core–shell structure [122] Design an ultrathin and flexible structure [123] To use single-electrode mode Use liquid metal electrode [124] Use of air-cushion mechanism [124]
Optimal locations for biomechanical energy harvesting	Center of gravity of upper body [125] Wrist movements [126] Knee movements [127] Feet motion [128] Legs and arms [113]	Movements of arms and legs [121] Human feet [129] Palms and fingers [130]	Relaxation and contraction of lung and cardiac muscles [131] Human skin [132] Clothes [133] Hand tapping [132]
Range of power output on nano-scale	0.5‒32 mW [113, 128, 134]	0.0002‒45.6 mW [121, 135, 136]	0.3‒4.67 mW [133]
Challenges	Difficult miniaturizing [137] Difficulties in integration [138] Design of flexible system [139]	Toxicity of piezoelectric materials Ultralow frequencies of human motions [140] Requirements of complex human movements [141] Rigidity and brittleness of Piezoelectric materials [142]	Need of surface modifications Humidity challenges The inflexibility of the electrode [143] Biocompatibility [144] Washability [122]

## Recent 3DP methods in fabricating NMEHs

Among various additively manufacturing approaches, as per ASTM F2792 standards, such as material extrusion, binder jetting, directed energy deposition, powder bed fusion, inkjet 3D printing, vat-photopolymerization, and laminated object manufacturing, the researchers have employed material extrusion and digital light processing (an advanced version of stereolithography) for fabricating 3DP-NMEHs. Recently, nanoimprint lithography has also been utilized for the low-cost fabrication of nano-devices [145]. Some optimal 3D printing methods, printing parameters, and 3D printers involved in fabricating 3DP-NMEHs are given in Table 8.

**Table 8:** Optimal 3D printing methods, printing parameters and 3D printers involved in fabricating novel structures of 3DP-NMEHs

Sr	Structure/shape	Printing approach	3D printer company	Printing parameters	Applications
1	Hierarchical and porous structures	FDM	HTS-300, Fochif Tech., pressure-controlled direct ink printer	Deposition speed of 2.8 mm s^−1^, extrusion speed of 0.008 mm s^−1^, filament diameter of 0.85 mm, and micro-nozzle diameter of 0.80 mm	Wearable electronics
2	Circular-shaped structures	FDM	Shining, Einstart-p, 3D printer	Uniform material extrusion from needle	Voiceprint recognition sensor
3	Square-shaped structures	FDM	30 M Hyrel 3D, USA 3D printer	Nozzle inner diameter of 0.5 mm	Self-healing/ stretchable conductor
4	Cubical shape	Hybrid UV based 3D printing	3D printer equipped with automatic UV curing, pressure-injection, and ink extrusion along with precision positioning platform	UV-based curing and printing precision of 1 μm	Ultra-flexible 3D printed TENG
5	Cylindrical structures	FDM	Makerbot Industry, USA, Replicator 2X 3D printer	Printing speed 90 mms^−1^ with plate temperature of 110 $^\circ{\rm C}$, using a raft to improve the adhesion between the plate and 3D printed parts	Noise-canceling
6	Hollow circular-shaped tubes	FDM	**–**	3D-printed circular tube of 1.1 cm inner dia and 1.2 cm outer dia	Human biomechanical energy harvesting
7	Lamellar porous constructions	DIW	**–**	An 840 μm dia cylindrical nozzle to print CNF ink through DIW printer	Multifunctional sensors
8	Biomimetic-villus shaped structure	DLP	Master Plus J 845 DLP printer from Carima, Korea	**–**	Dust filter
9	Grating disk-like structure	FDM, SLM	ProX DMP 320 from 3D Systems, metal 3D printer	3D printing in argon gas with a 245 W laser, with 60 μm layer thickness, 82 μm side step, and 1250 mm s^−1^ mark speed	Sustainable energy harvesting
10	Sponge	FDM	Z300, Beijing Huitianwei Technology Co., Ltd, China, 3D FDM printer	Nozzle size of approx. 10 μm	Energy harvesting applications
11	Zigzag design	FDM	FDM printer Z300, Beijing Huitianwei Technology Co., Ltd, China	Material extrusion with 0.1 mm layer height	Mechanical energy harvesting
12	Hierarchical morphological structures	DIW	**–**	Parallel printing direction with 0.2 mm tip diameter	Mechanical energy harvesting

The references of the research papers cited in this table are provided in the Additional file 1

*FDM* fused deposition modelling, *DIW* direct ink writing, *SLM* selective laser melting, *UV* ultraviolet

### Material extrusion

The material extrusion, also known as fused deposition modelling (FDM), has been widely used to fabricate 3DP NMEHs due to its high speed, cheaper materials, large-volume printing capability, and a wider range of functional materials. Moreover, FDM facilitates faster solidification, the usability of various thermoplastics, exceptional chemical characteristics, easy removal of supporting members, excellent heat resistance and high mechanical strengths. Shihua et al. [146] used the material extrusion to develop spongy micropatterns on TENG films made of polyamide and PDMS polymers. It was revealed that the FDM-based fabrication of thermosetting materials, for instance, PDMS, was easier than other printing approaches. The TENG was developed in the following stages: firstly, a CAD model made by Siemens PLM Software, Unigraphics NX 10, was sliced through a Cura, Ultimaker software. The required surface morphologies and properties were analyzed to develop the machine code and loaded into the 3D printer (version Z300, Beijing Huitianwei Technology Co., Ltd, China). The 3D printed specimen was 120 mm long, 100 mm in width, 0.1 mm in height, and 0.3 mm thick. The sponge-like structure made through FDM was characterized by a larger contact area and induced charge.

**Fig. 13:**
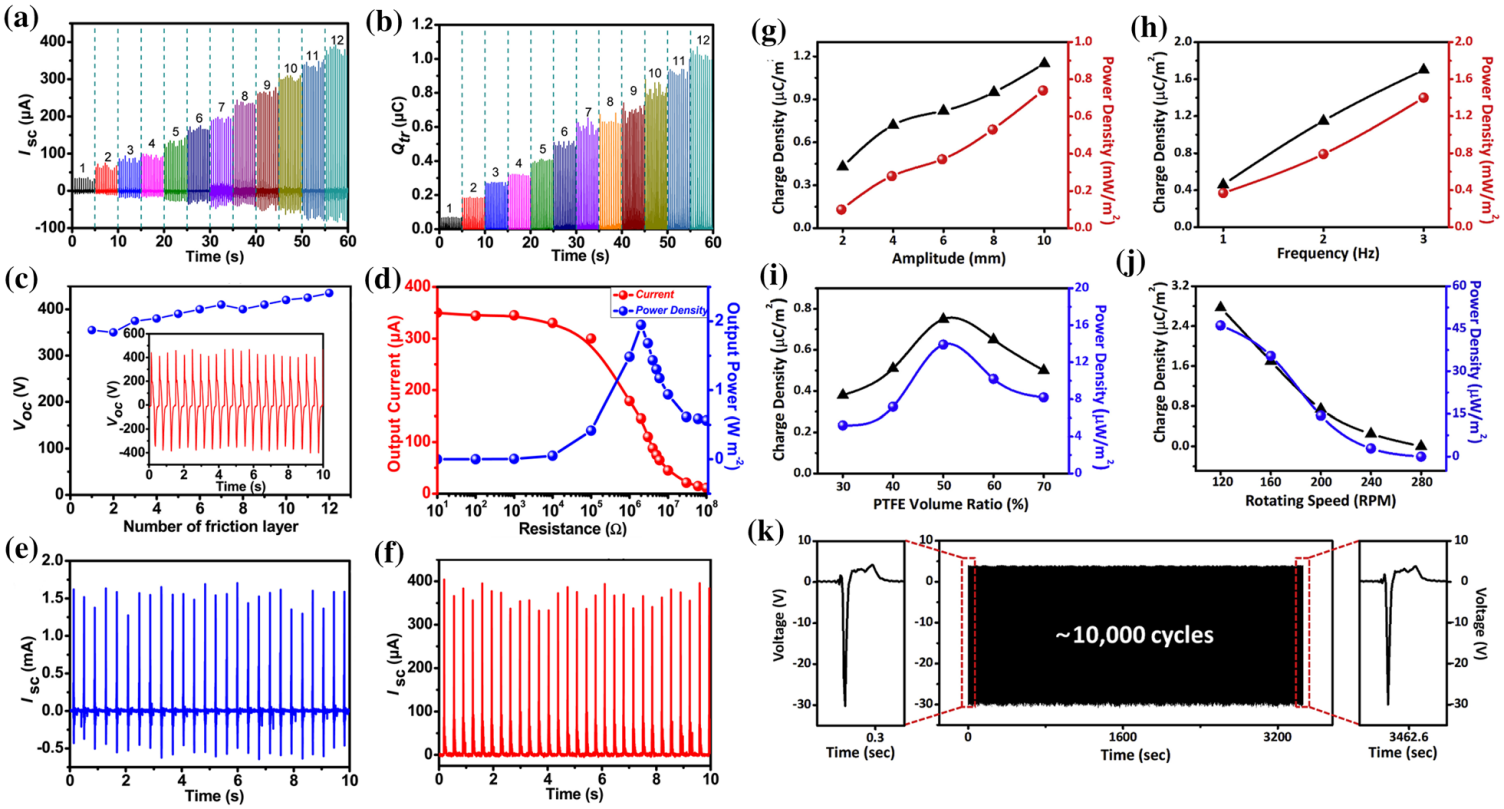
Output performance characteristics of the 3DP-TENG proposed by Tian et al. [147]: **a** short-circuit current, **b** induced charge **c** open-circuit voltage with twelve different pairs of fraction layers, **d** output current and power density with the external resistance **e**, the transformed short-circuit current, and **f** rectified short-circuit current [147]. Electrical performance of the 3DP-NMEH proposed by Hong et al. [148] under various test conditions: The induced charge density and powder density under the vertical operational mode and various: **g** amplitudes (2–10 mm) **h** frequencies (1–3 Hz), charge and powder densities achieved by the device under rotational working mode and various: **i** Teflon volume ratios (30–70%) **j** speeds (120–280 RPMs) **k** durability test under vertical operational mode over 10,000 operational cycles [148] (Images are re-used with the permission of the publisher)

Similarly, M. Tian et al. [147] demonstrated the fabrication of thermoplastic elastomer filaments on thin copper films by FDM printing. The Cu films were integrated with the polytetrafluoroethylene film to enhance the nanodevice’s structural resilience and increase the triboelectric charge accumulation. The proposed device exhibited a short-circuit current of 375 μA and a maximum power density of 2 Wm^−2^ and could efficiently power the EF pollution degradation system. The output performance of the 3DP-NMEH in terms of accumulated charge, short-circuit current, open-circuit voltage, and output power are shown in Fig. 13a–f. It was revealed that a higher short-circuit current and induced charges were achieved with increasing friction layers on the triboelectric material. Connecting the friction layers in parallel caused a significant increase in open-circuit voltage.

### Digital light processing (DLP)

DLP, the advanced development of stereolithography, employs a light projector for photoreactive curing of the material and has been excessively utilized for precise printing of the resins onto the 3DP-NMEHs substrates. DLP-based printing facilitates fast solidification of resins upon exposure to bright light. Moreover, it consumes less material, minimizes waste, and eliminates the additional use of powder [9]. Hong et al. [148] demonstrated applying a DLP Master Plus J 845 machine to print a commercially available ABS photopolymer onto a Teflon film. The cylinder-shaped structures of 38 mm height and 42 mm diameters were developed consequently. A silver paste was adsorbed on the outer surface of the ABS substrate, and a dust filter was also demonstrated. The unique structure made by DLP facilitated a smooth flow of the air through the device’s proximity. The proposed 3DP-NMEH device generated a peak power density of 1.4 mW/m^2^ in vertical operational mode and 13.9 μW/m^2^ in rotational mode. The device’s output performance optimization for the vertical and rotational working modes is shown in Fig. 13g–k. In the vertical operational mode, with an increase in the amplitude along the z-axis, an effective increase was observed in the charge and power densities of the device, making the freestanding working mode more feasible for optimal energy harvesting. On the other hand, the output current increased directly with the input excitation frequencies. The output power was enhanced at the PTFE powder’s 50% volume ratio in the rotational working mode.

### Benefits and drawbacks related to 3D printing of NMEHs

The benefits and drawbacks of 3D printing of nano-mechanical energy harvesting systems are presented in Table 9.

**Table 9:** Benefits and challenges related to 3D-printing of the nano MEH systems

Benefits	The accurate pattern-making ability for architectural customization of the nanogenerators. The tiny-structured patterns allow easy implementation and mechanical resilience to electronics
	Lower power consumption and environmental impact. Facilitates long-term sustained production
	Provides faster speed and high-fabrication compatibility
	Less human intervention is required in the printing of the parts and post-treatment
	Abatement of waste materials and overall material usage
	3D printing is a safe, sensitive, and flexible fabrication facility
	Excellent chemical attributes characterize the printed objects
	FDM printing gives heat resistance and good mechanical strength to the prototypes
	Facilitates easy and manual removal of the supporting elements, i.e., water-soluble wax at the end of the printing process
	Fast solidification of the printed components on various substrates
	Exceptional printing accuracy and resolution
Limitations	The complex integration of functional polymers
	To achieve and maintain the tiny gap required between triboelectric polymer surfaces is challenging
	The formation of precise macro/nano dimensional architectures is yet to be studied
	The combination of various materials for 3D printed TENGs is problematic
	The development of integrated systems comprising of nanogenerators and functional devices and simultaneous handling of multiple printing materials is still challenging
	3D printed nano MEH systems are needed to be biocompatible and integrated with biological tissues
	End-of-life recyclability/biodegradability of the 3D-printed components is a critical concern to reduce impacts on the environment and the human body
	For complex models, the printing time is often high
	Digital light processing (DLP) demands more light sources, for instance, arc lamps during printing
	A liquid crystal display is employed at the entire 3D-printed deposit during a single layer of the DLP
	In 3D printing of fabrics, it is challenging to develop appropriate CAD modeling in order to facilitate the simulation of the draping of the textile across a curved surface
	3D-printed textiles are not strong enough and tend to break easily due to the conventional textiles' lesser flexibility. Hence, they are not very suitable for day to day textile applications

The references of the research papers cited in this table are provided in the Additional file 1

The commonly achieved length scales, capacities, and printing techniques currently observed in rapid prototyping of nano-mechanical energy harvesting mechanisms are enlisted in Table 10.

**Table 10:** The capacities, length scales and printing methods of recently demonstrated 3DP-NMEHs

Sr. No	Size/Length scale	Printing technique	Output performance			References
			Current (μA)	Voltage (V)	Power (W/m^2^)	
1	3 cm × 3 cm	FDM	6.14	306	~ 237	[146]
2	3 cm × 3 cm	DIW	190	170	~ 185	[149]
3	**–**	DLP	2.3	**–**	~ 46	[148]
4	**–**	FDM	0.9	90	~ 45	[150]
5	**–**	FDM	0.26	0.1	~ 0.5	[24]
6	4 cm × 3.5 cm	FDM	375	410	~ 2	[147]
7	4 cm × 1.5 cm	FDM	7.6	103	~ 7	[151]
8	3.2 cm × 3.2 cm	DIW	0.94	55.8	~ 29	[152]
9	3 cm × 3 cm	FDM	4	100	~ 40	[153]

### Optimal 3D printing methods for NMEH functional parts

Seol et al. [25] developed 3D printed electrodes, casing, and triboelectric parts for a sustainable energy device that could implement an ideal resource utilization. The Titanium Grade 23 powder was used to 3D print metal blades for a triboelectric nanogenerator using a ProX DMP 320 metal 3D printer from 3D Systems. The printing was optimally accomplished using an 82 µm sidestep, 1250 mm/s speed, 60 µm layer thickness under an argon environment, and laser power of 245 W. The printed blades were thermally treated to relieve internal stresses and prevent surface oxidation using a 1216 FL CM furnace with a Furnace 3504 temperature controller in an inert environment and an argon flow rate of 30 psi. Finally, the heat-treated metal blades were processed through a Millport 2 milling machine equipped with a Walter F4033 Milling Cutter to obtain a smooth surface. The blades were detached from the base using an electrical discharge machine. The all-3D-printed TENG could generate an RMS short-circuit current of ~ 19 μA, the open-circuit voltage of 231 V, and power of 2.13 mW and be demonstrated as a sufficient power supply for wireless electronic sensors.

Yang et al. [154] demonstrated a composite piezoelectric material made of Poly (vinylidene fluoride) (PVDF) combined with barium titanate BaTiO_3_ and coated with carbon nanotubes (CNT). The CNT-coated PVDF/ BaTiO_3_ composite powders were prepared using a complex chemical process [154] and printed through an SLS printer to develop piezoelectric material. The printing parameters, including laser power of 40 W, the beam diameter of 200 μm, a laser scanning speed of 9600 mm/s, laser scanning distance of 0.3 mm, and powder layer of 0.1 mm, were used for SLS printing. The demonstrated PENG could generate a piezoelectric output of 19.3 V and 415 nA, which was sufficient to charge a 1 μF capacitor to ~ 5 V within 3 min.

In another study conducted by Zeng et al. [155], the Mask-Image-Projection-based Stereolithography (MIP-SL) was used to 3D print a honeycomb-structured BaTiO_3_-based PENG for ultrasound sensing. The photocuring was induced using an LED-based digital light projector with visible light of 405 nm wavelength. From the experimentation, the optimal exposure time was determined to be 37 s per layer, with a thickness of 30 μm for each layer. Finally, the printed samples were sintered for 4 h at 1350 °C to get dense ceramic parts. The printed sample of the piezoelectric nanogenerator achieved a piezoelectric constant of 60 pC/N and an output voltage of 180 mV. Han et al. [26] developed a 3D printed wind turbine using a desktop 3D printer, MakerBot Replicator 2. The turbine blades, end plates, supports, and base were 3D printed, and the tests were conducted in a wind tunnel. The prototype could achieve a maximum overall energy conversion efficiency of ~ 6.6% and electrical power of 0.31 W.

### 3D printing methods for energy harvesting fabrics

Energy harvesting fabrics are conventionally made with powder bed fusion or material extrusion processes. Kyttanen et al. [156] revolutionized the 3D printing of energy fabrics by projecting a textile pattern on a piece of clothing and thereby generating a 3D CAD model of the complex pattern resembling a chainmail structure. After that, such textiles were extensively printed with FDM and SLS. In another study [157], unique SLS and FDM techniques were demonstrated for fabricating weft-knitted single face textile structures. FDM printing was used to make lace patterns, and it was found that hard polylactic acid (PLA) and ABS were too brittle for the required textile structure, whereas soft PLA was suitable for it.

One of the significant challenges confronted by fabric 3DP is modelling and simulation of fabric draping around the curved surface. Bingham et al. [158] developed a CAD model for a complex 3D conformal textile using which the world’s first 3D conformal seamless rapidly prototyped textile fabric was produced through SLS printing. However, the conventional CAD software was not equipped with the capability to model complex curved textile structures. Therefore, it was a pretty tedious and time-consuming process. To deal with it, Bingham et al. [158] introduced a specialized textile CAD module based on the Representative Volume Element (RVE) concept to model printable textiles.

Furthermore, they demonstrated a customized TexGen package that could use an FEA mesh imported from the conventional software. Another study [159] made efforts to generate STL data to manufacture conformal 3D printed textiles efficiently. David [160] demonstrated a classified digital code for 3D printing of three major textile structures: woven, linked and knitted.

Most printed textiles have been manufactured using nylon, PLA, ABS, acrylic, and polyurethane. The most influential factors, including textile structure, material, and process, are currently significantly involved in the 3D printing of energy harvesting fabrics. It is crucial to develop new materials with optimal properties for the 3D printing of energy harvesting fabrics. Similarly, developing specific rapid prototyping techniques compatible with material properties and novel textile structures is another challenge. Besides the issues related to alternative re-meshing, mesh confirming, and CAD modelling of complex surface topology need to be studied to enhance the 3DP capabilities for EH fabrics. Mueller et al. [161] demonstrated that the selection of printing materials for MEH textiles should be driven by their stiffness, ductility, service loading conditions, flow characteristics through the nozzle, and melting temperature, in addition to the structure and type of the desired products. For motion-induced energy harvesting textiles, the decisive characteristics of the material should be durability, strength, flexibility, extrusion, triboelectricity, and piezoelectricity. The polyvinylidene fluoride (PVDF) fibers and their blends with various nanomaterials are extensively used for their diverse multifunctionality.

## Emerging applications and implementation of 3D-printed nano MEH systems

**Fig. 14:**
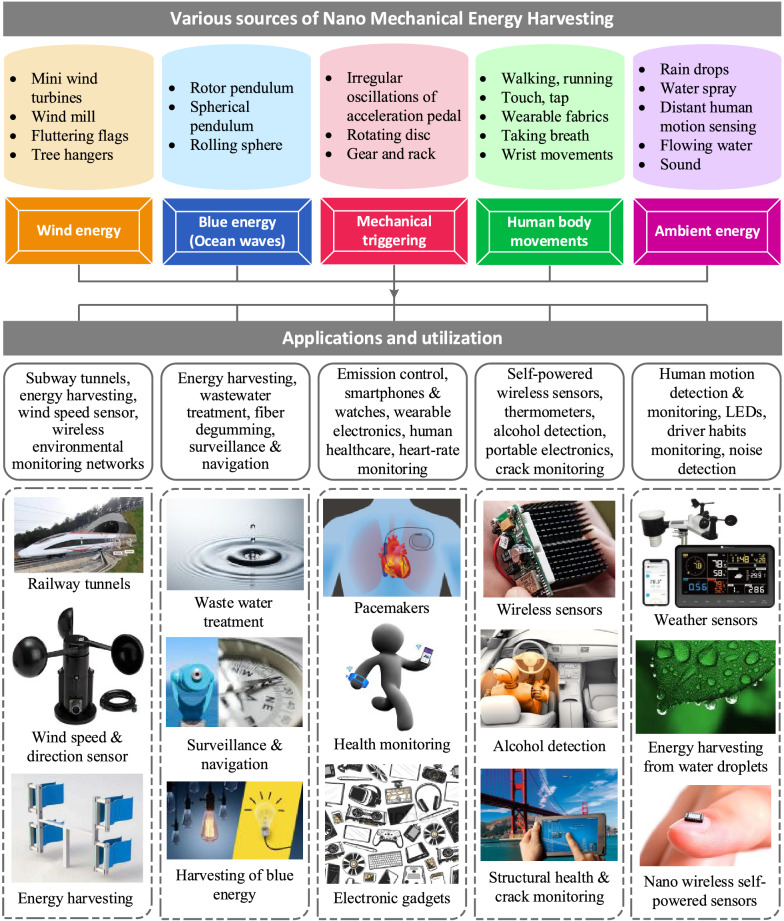
Emerging applications of nanomechanical energy harvesting systems based on various sources of energy

Figure 14 highlights various renewable energy sources widely used to harvest mechanical energy through 3D printed NMEH systems and the potential applications for each category. These sources are wind, ocean waves, biomechanical energy, raindrops, sound, and ambient vibrations (such as railway track vibrations, wind swirls in a railway tunnel, and triggering of acceleration paddle). The major applications include optical sensing [162], low-power soft electronics [163, 164], pressure/strain sensors [165], self-powered wireless monitoring networks [166], wearable sensors [167], actuators, off-shore electronic appliances, low-power personal electronic gadgets, mobile phone batteries, wireless Bluetooth hand-free, watches, water desalination [168], monitoring systems installed in high-speed railway tunnels [169], water splitting [170], structural health monitoring [171] and heat or cold detecting devices [172]. Other applications are wireless temperature sensor nodes [173], commercial LEDs, wearable health monitoring devices [174], and weather sensors. In addition, some non-conventional and advanced applications are discussed hereafter.

### Self-powered electronic sensors

**Fig. 15:**
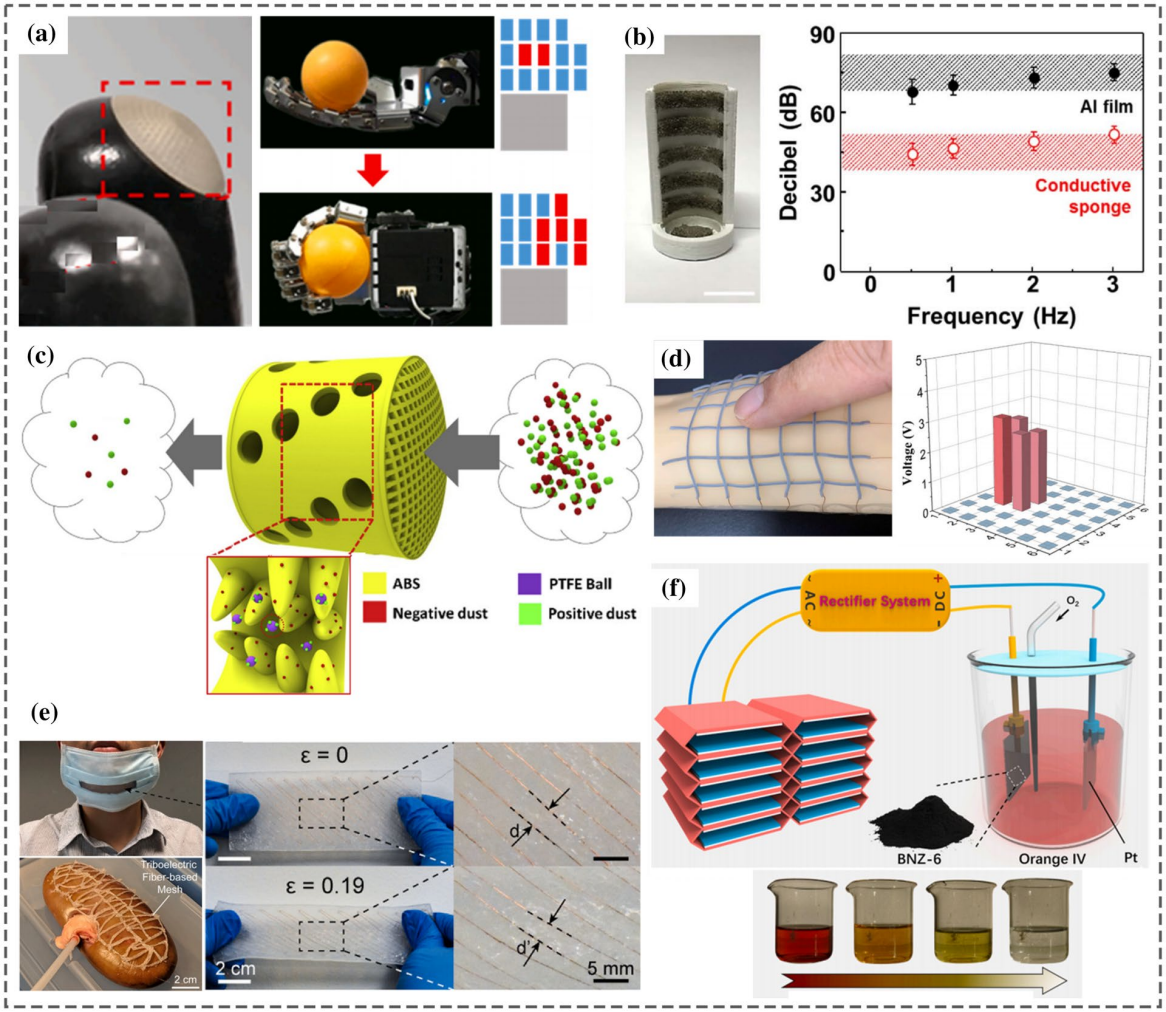
Emerging applications of 3D-printed nano-MEH systems **a** conformal strain sensors attached on robotic hand showing the holding and gripping of a ball [175] **b** 3D printed noise-cancelling TENG causing a reduction of approximately 20 dB in the noise level of a normal conversation [150] **c** a 3D-printed biomimetic villus structure acting as an eco-friendly dust-adsorption system [148] **d** a 3D printed self-powered e-skin with meshed structure and corresponding 3D output voltage signal [176] **e** a human wearing an orthogonally stretchable triboelectric facemask and 3D-printed TENG fiber-based mesh of a kidney-conforming sensor [177] **f** a self-powered pollution degrading electro-Fenton (EF) system used for discoloration processes of the organic pollutant (Orange IV) solutions [178], (Images are re-used with the permission of the publisher)

Nanogenerators have been widely used as self-powered electronic sensors. For example, Zhang et al. [175] incorporated boron nitride nanotubes (BNNTs) to develop a nanofiller-polymer piezoelectric composite. The microstructured piezoelectric composites revealed an excellent relative sensitivity of 120 mV/(kPa⋅wt%) under a load of 1–400 kPa, which was tenfold higher than unmodified BNNTs. Furthermore, the 3D printed piezoelectric composites were successfully demonstrated as a self-powered conformal tactile sensor array that could be potentially used for haptic sensing of the robotic hand and detection of the spatial distribution of forces on irregular surfaces. For example, the conformal strain sensor integrated into a robotic hand to grip and hold a ball is shown in Fig. 15a.

Similarly, a transparent triboelectric device with improved structure robustness and enhanced performance self-recoverability was fabricated through 4D printing without any molds. The device had a maximum power density of 56 mW/m^2^ and was demonstrated as a self-powered sensor to detect human joints' bending angles. The self-recoverability due to the thermal treatment of shape memory polymer and 4D printing may offer great potential in developing self-powered sensors for robotic control and sensing in precise and sophisticated structures. Using 3D and 2D printing, an all-printed nano-cellulose paper-based TENG was characterized by enhanced abrasion durability and high output power. The device could be printed anywhere, making it remarkably achievable in remote locations, where the logistics are expensive and complex, including the International Space Station, artificial satellites, and other planets.

### Noise-cancellation

A high output-power 3D printed triboelectric nanogenerator was applied for energy harvesting under a harsh environment and noise cancellation of the MEH device for a stable, long-lasting operation [150]. The TENG was comprised of a fully-packed, cylinder-shaped structure with a linearly patterned aluminum film on the inner surface having polydimethylsiloxane bumpy balls inside the device. The design was optimized to increase the output power up to 45 mW and charge a smartwatch battery. The operation noise was reduced by approximately 20 dB (to the noise level of ~ 50 dB) without any output power degradation using a polyurethane sponge embedded with conductive and highly compressible silver nanowires, as shown in Fig. 15b. It was revealed that the output performance of the MEH device was not decayed even after it was immersed in the water. The noise produced during the operation was ranged from 45 to 52 dB, equivalent to the noise level of a normal conversation.

### Eco-friendly dust-adsorption system

A 3D-printed triboelectric nanogenerator with a biomimetic villus structure [148] has been developed to demonstrate an eco-friendly dust-adsorption system. The rotational-direction mode and vertical-direction mode were employed to achieve a fourfold and fivefold increase in the output power with Polytetrafluoroethylene powder as the triboelectric material. A large electrostatic charge induced between PTFE powders and an acrylonitrile butadiene styrene surface was used to efficiently absorb the dust particles of various sizes (Fig. 15c). Hence, an eco-friendly dust-filtration system was designed. It was revealed that the dust filter was easily reusable and stable due to the application of the PTFE powers and the polymer-based ABS. The filtration efficiency of the dust-filtration system was evaluated to be 41% which was not influenced even after washing. Experimental testing affirmed that an efficient filtration of ultrafine dust with a diameter of < 2.5 μm was achievable at a rate of approximately 40% over 75 min. The study justified the application of 3D printed nanogenerators as energy harvesting and self-powered dust-filtration systems.

### Self-powered e-skin for wearable electronics

The stretchable 3D printed smart textiles have been established as an effective source of power for wearable electronics. Based on the triboelectric effect, 3D printed stretchable elastic fibers with a coaxial core-sheath structure were prepared using PTFE particles and graphene [176]. The e-skin consisted of a conductive core and insulative sheath with enhanced rheological characteristics. The structure can be used as wearable tactile sensors (or e-skin) due to matrix tactile sensing ability through the interlaced structure of weft and warp, as shown in Fig. 15d. In addition, the enhanced physical and chemical properties such as breathability, washability, robustness, and super stretchability made it promising for wearable electronic applications.

### Biomedical monitoring and speech recognition

Recently 3D printed triboelectric stretchable structures were developed for applications related to biomedical and human activity monitoring. The maximum output power density of 31.39 mW/m^2^ was generated by the single 3D-printed elastomeric metal-core silicone-copper fiber [177]. The demonstrated applications of the proposed prototype were wearable mechano-sensors for organs, monitoring of perfused organs, and speech recognition (or silent speech) in the absence of sound generation by the human subject. The 3D-printed stretchable membranes and form-fitting meshes were combined with machine-learning algorithms of signal processing for real-time monitoring of perfusion-induced kidney edema, as shown in Fig. 15e. Furthermore, the device was modified for speech recognition with a word classification accuracy of 99% in human subjects' absence of sound production, which justifies using 3D-printed triboelectric NMEH devices for self-powered sensing applications in medicine and biomanufacturing.

### Electro-Fenton (EF) pollution degradation systems

Figure 15f shows the application of a self-powered electro-Fenton (EF) degradation system based on triboelectric effect in the discoloration processes of orange IV organic pollutant solution as an energy-saving and environmentally-friendly approach to pollution degradation [178]. The multi-layered TENG-based flexible prototype was fabricated through 3D printing and comprised the N-doped carbon cathodes acting as electro-Fenton catalysts. As a result, 95% and 96% of the degradation efficiencies were recorded experimentally for violet and orange IV pollutant solutions, respectively, in a 60 min operation. Similarly, a flexible 3D printed electro-Fenton degradation device was reported based on a self-powered nanogenerator to eradicate methylene blue using cathode catalysts made of biomass-based carbon materials [147].

### 3D activity inertial sensor for healthcare telemedicine applications

**Fig. 16:**
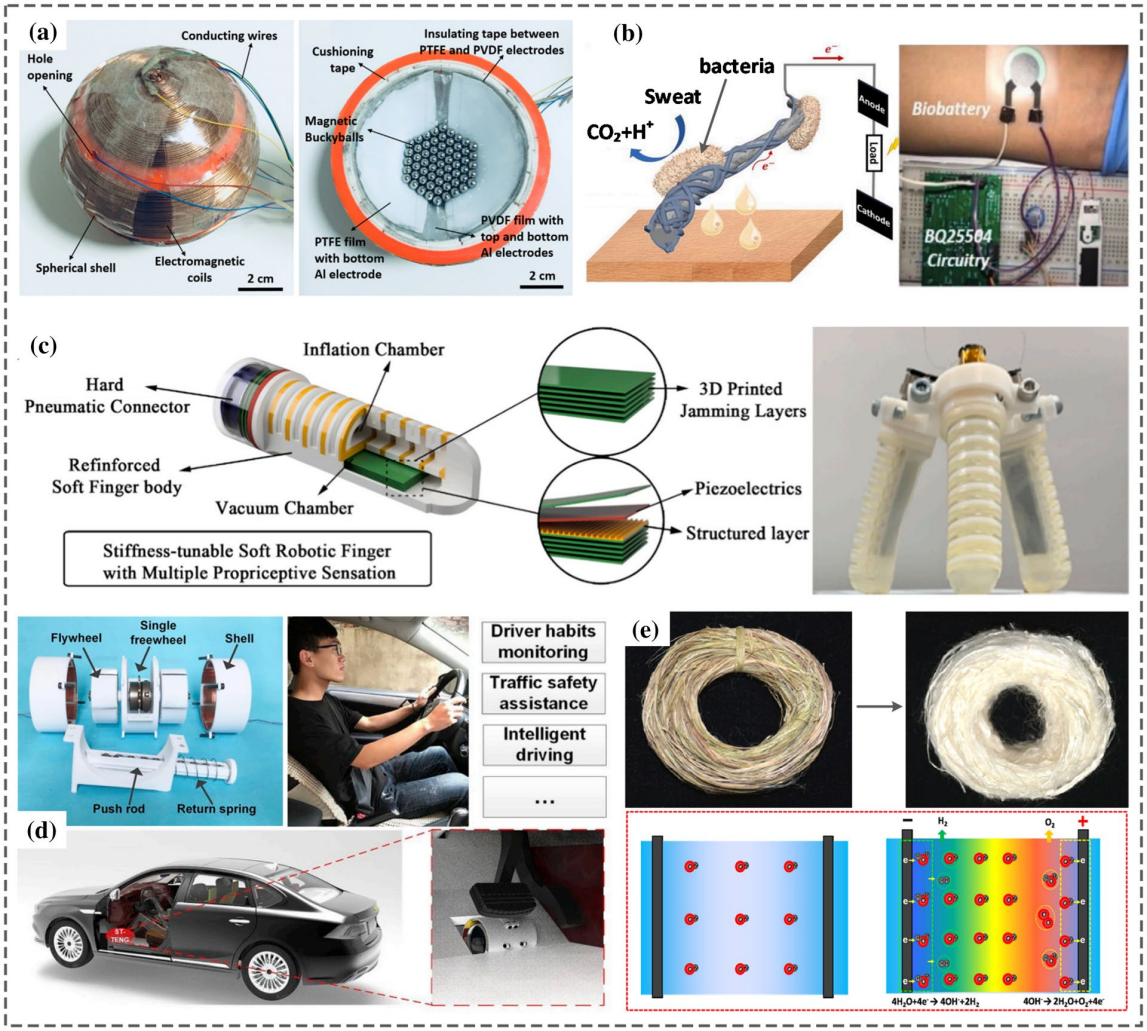
Emerging applications of nano-MEH systems **a** a self-powered 3D activity inertial sensor (3DAIS) used for multi-axis rotation and acceleration sensing [37], **b** schematic of the bacterial energy harvesting from human sweat [179], **c** a soft robotic finger with sensing/jamming multi-functional structures and a robotic gripper for manipulation and grasping [180] **d** the driver monitoring TENG and installation of the EH device in a car [181] **e** application of TENG for the transformation of raw ramie fiber into the degummed fiber and use of electric field provided by TENG for wastewater treatment [182], (Images are re-used with the permission of the publisher)

As shown in Fig. 16a, a hybrid energy conversion model based on electromagnetic, triboelectric, and piezoelectric nanogenerators was demonstrated as a self-powered three-dimensional activity inertial sensor (3DAIS) [37] for multi-axis rotation and acceleration inertial sensing. The proposed device consisted of magnetic buckyballs captured inside a 3D–printed spherical casing. The inner walls of the shell were deposited with layers of aluminum, PTFE, and PVDF films, whereas wire coils surrounded the outer surface. The sensor performed effectively in state monitoring of human activities and motion recognition applications. Moreover, the 3DAIS could sense the x, y, and z components of the acceleration during linear motion and yaw, roll, and pitch components of angular velocity during rotational motion. Furthermore, due to its hybrid EMG-TENG-PENG nature, the device was capable of harvesting energy from various sources such as rotations, human movements, and 3D vibrations, making it a self-powered dual-purpose NMEH gadget. Other potential implications of the 3DAIS include advanced motion sensing systems, self-powered wearables, and healthcare telemedicine applications.

### Electricity generation from sweat-eating bacteria

Harvesting biomechanical and thermal energy from human motion and body heat are conventional methods. Recently, nano-energy harvesting systems have been employed to generate electricity from human sweat-eating bacteria. Human skin is acidic and can be used as a potential energy source. Therefore, an innovative and long-standing approach was demonstrated to extract energy from human sweat for stand-alone and self-sustainable electronic-skin systems. The potential applications include security, fitness, healthcare, and environmental monitoring sensors. The 3D printed PDMS-based microfluidic device was fabricated that uses metabolisms of sweat-eating bacteria existing on sweaty skin [179], as shown in Fig. 16b. These bacteria include Staphylococcus capitis, Micrococcus luteus and Staphylococcus epidermidis. A microbial fuel cell also referred to as biobattery, uses bacteria as a biocatalyst and converts the chemical energy of human sweat into electrical energy. The skin-mountable NMEH can be employed as an integrated battery-free skin-interfaced energy harvesting system for various suggested applications.

### 3D printed piezoelectric soft robotic gripper

Figure 16c shows the application of a novel flexible 3D printed NMEH system for soft robotic grippers to execute dexterous and safe grasping of the objects. The device was fabricated by a multi-material 3D printing technique with a polyvinylidene difluoride layer and a microstructured jamming system and demonstrated as a self-powered, multifunctional sensor. The sensor integrated with the soft finger facilitates passive proprioceptive sensations of stiffness and curvature with optimal sensitivity of 0.09 Vm/N and 0.55 mV m, respectively [180]. Furthermore, the sensor can also control finger stiffness within a range of 15–44 N/m without disturbing the system’s dynamics. In addition, a three-fingered robotic gripper was successfully tested to measure finger stiffening and bending in the pick and place method. Significantly the application reveals the potential of achieving anthropomorphic grippers.

### Driver habits monitoring

As shown in Fig. 16d, a triboelectric nanogenerator was employed to monitor the drivers’ habits on the road. As a social concern, driver habits are directly related to traffic safety, making it inevitable to monitor driver habits during vehicle driving. The proposed triboelectric nanogenerator comprising the outer shells, a push rod, a freewheel, and two flywheels were installed beneath a car’s acceleration pedal [181], as shown in Fig. 16d. The TENG was designed to harvest energy from the random triggering motions of the driver as a result of the driver’s step movements. Moreover, the device records the varying driver’s triggering motion patterns and characterizes the driver’s habits. Experimental investigation revealed that a short-circuit current of 15 μA and an open-circuit voltage of 400 V could be achieved. In addition, the recorded data could be used to monitor road conditions and driver habits. Thus, the proposed system highlights the potential to develop an intelligent driving system.

### Fiber degumming, wastewater treatment, and textile applications

Degumming is an essential process used to reduce wrinkles and hold the shape of the ramie fiber. In addition, it attributes a silky glow to the fabric. A novel NMEH system based on a water-driven triboelectric nanogenerator was employed for ramie fiber degumming and harvesting energy from the water ripples [182], as shown in Fig. 16e. Experimental investigation revealed a significant enhancement in the degumming efficiency. Moreover, the quality of the resulting fiber was significantly improved in terms of mechanical properties and surface morphology. The NMEH based degumming provides a sustainable approach to separate the non-cellulosic structures from ramie fibers and reduces the reliance on conventional chemicals used in the traditional degumming methods. Furthermore, the proposed self-powered NMEH was used to electrochemically degrade the polluted degumming wastewater using the harvested energy from flowing wastewater. The water treatment unit produced 3.5 mA and 10 V and cleaned up to 90% of the pollutants in the degumming water in 120 min. Empirically, the proposed NMEH was characterized as a cheap, environmentally friendly, and highly stable technology for ramie fiber degumming and wastewater treatment with high degradation and degumming efficiency and could be potentially employed as a sustainable approach in textile industries.

## Challenges and future perspectives

**Fig. 17:**
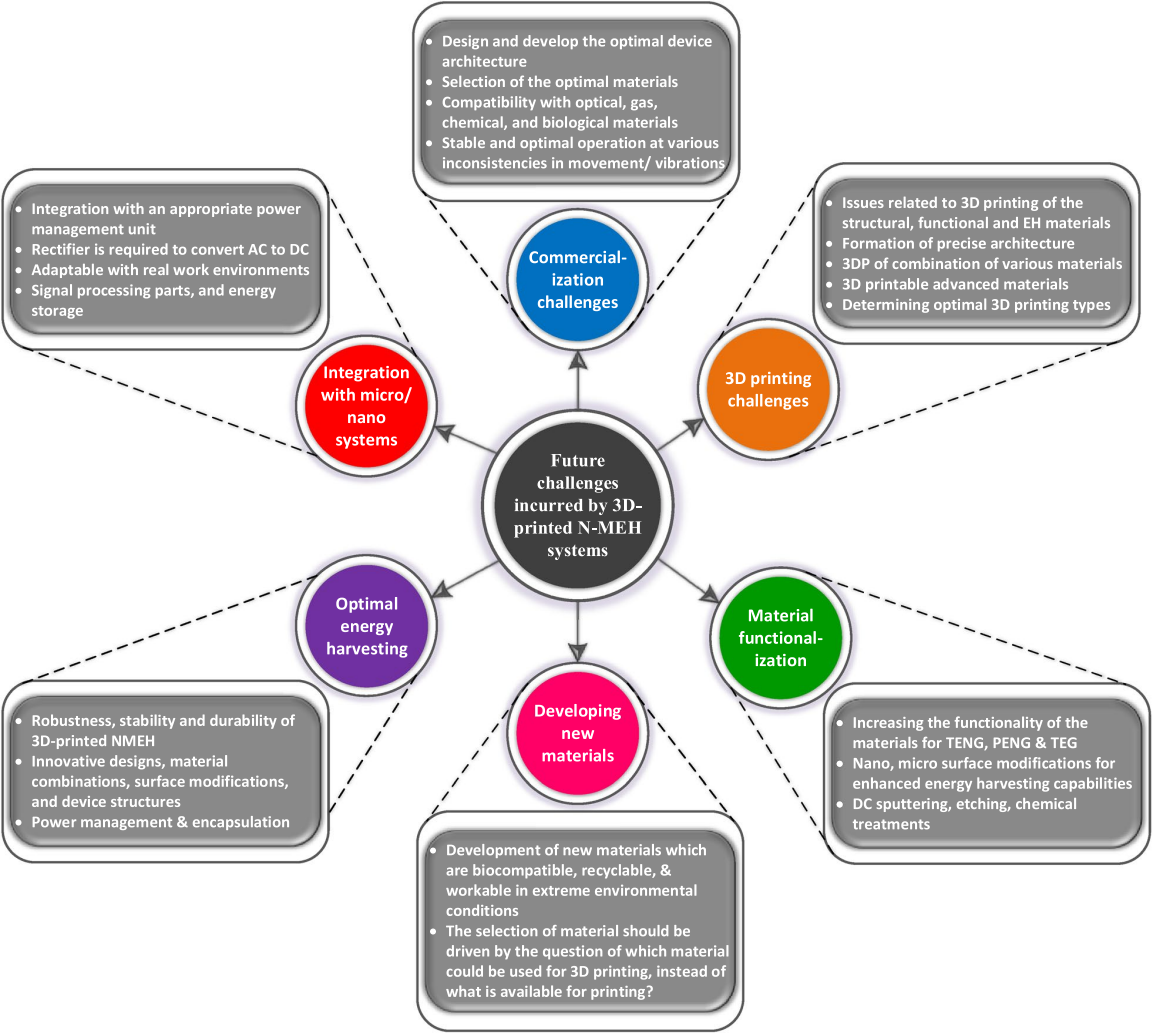
Challenges and future perspectives incurred by 3D-printed NMEH systems

As an advanced research domain, the 3D printed nanomechanical energy harvesting systems are confronted with many challenges related to 3D printing, material functionalization, development of advanced materials, optimal energy harvesting capabilities, commercialization, complex power management, hybridization, load electronics, device structure, stability, and accuracy. This section briefly summarizes the challenges and future perspectives of 3D printed NMEH, as shown in Fig. 17.

### 3D printing of MEH and material functionalization

For the development of nanostructured functional components for nanogenerators, recently the 3D-printing technology has gained significance over typical methods such as reactive ion etching, spin coating, electrospinning, and imprint lithography [183] due to the advantages of efficient manufacturing, low cost, adaptability, speed, sustainability, precision, and durability. However, despite the several benefits mentioned above, there are many technical challenges incurred by 3D-printing technology to develop NMEH systems. For example, although digital light processing (DLP) and fused deposition modeling (FDM) have been extensively used to modify surface morphologies of the NMEHs, the printing of polymeric resins to develop patterns < 100 μm in size is challenging [184]. Therefore, efforts are required to study 3D printing to modify the surface morphology of the materials for optimal nanopatterning to improve the surface work function, triboelectric coefficients, output power, and contact area. Furthermore, most 3D-printable polymers cannot fulfill the requirements for projected implementation in biomedical implants and wearable electronics. Hence, the critical problems related to 3D printing in functionalizing piezoelectric, thermoelectric and triboelectric materials for these applications need to be addressed. Furthermore, concerning the implementation of printed nanodevices inside the human body, encapsulation should be biocompatible and connectable with biological tissues. Furthermore, for designing the contact-separation mode of the 3D printed triboelectric devices, the significance of the mechanical resilience of the device structure should not be ignored.

In most studies, only the material extrusion and digital light processing approaches have been employed to develop 3DP-NMEHs. Applying other 3D-printing techniques such as SLM, SLA, and SLS to develop nanogenerator structures and materials is crucial. Secondly, the challenges related to 3D printing of integrated NMEHs (nanogenerators and functional accessories such as implants, sensors, and actuators) must be considered. For instance, recently, 3D-bioprinting has been widely employed to print artificial human tissues and organs. As a solution to the problem, the biocompatible nanogenerators and biomedical implants can be printed all together to eliminate the subsequent integration.

3DP based manufacturing of conventional MEH devices is challenging due to the unavailability of favourable printing processes and materials. For example, 3D printing of the coil spools and permanent magnets used in electromagnetic generators is not achievable currently. In addition, due to several factors such as low output voltage, high output current, unavoidable coil losses, and the requirement of bulky gear mechanisms for enhancing speed to maximize output power levels add to the difficulty of 3DP-based development of micro/nanoscale EMG-based MEH devices for the human body applications.

Similarly, using 3D printing to develop rare-earth dielectric materials and their high-temperature processing for piezoelectric nanogenerators (PENG) is complicated and needs to be addressed. Moreover, inkjet printing of various functional ceramic materials for PENG devices is challenging due to the unapproachability of a specialty printer head. In this regard, significant efforts are needed to develop 3D printing strategies and novel materials for fabricating conventional MEH devices. Triboelectric nanogenerators are highly compatible with 3D printing technology; however, it is restricted by the wear of the modified surface morphology caused by friction and adhesion of the printed nanopatterns.

### Development of new materials

Currently, most of the 3D printed NMEHs are manufactured using photo-acrylic, nylon (PA), acrylonitrile–butadiene–styrene (ABS), polylactic acid (PLA), and polyurethane. Potential challenges include developing new smart materials and enhancing their properties for 3D printing of miniaturized electromagnetic, triboelectric, piezoelectric, thermoelectric energy harvesting devices, fabrics, implants, and self-powered sensors. These materials should be biocompatible, recyclable, and workable in extreme environmental conditions. Also, the development of specific 3D printing approaches driven by the structure of the NMEHs, and the nature of the materials is crucial. The 3D printing materials used in piezoelectric and thermoelectric devices are complex and need many chemical procedures to be developed. Therefore, the development of easily manufacturable materials could be appreciable. Nanostructured piezoelectric materials such as PZT, BaTiO_3_, ZnO, PVDF, InN, GaN, and CdS have been demonstrated frequently as efficient and effective building blocks for converting ambient mechanical energy into electricity. However, the problems associated with these materials are that (1) most of them are pretty brittle, (2) work only at insignificant levels of strain (**~ **1%), (3) have lower densities of output power, and (4) have quite tedious manufacturing processes. Henceforth, recently micron-sized fiber-based electrical power generators and bio-templated nanomaterials [185] are demonstrated for harvesting energy from ambient environments. These textiles are lightweight, comfortable, and comparable to conventional fabrics in strength, quality, and aesthetics. To sum up, the selection of materials and the 3D printing process should primarily be driven by what type of material could be used for 3D printing instead of what is available for printing.

Regarding heat EH devices, the temperature difference ($\Delta T)$ between the human body and the ambient environment is mostly near 20 °C, which can be considered as ease for 3D printing of thermoelectric (TEGs) and pyroelectric (PEGs) generators and selection of printable materials. However, under smaller $\Delta T$ values, not much energy can be expected from TEGs and PEGs. Therefore, selecting materials with greater energy conversion efficiencies under ambient temperatures is crucial to achieve optimal device performance. Finally, the curved surface of human skin requires flexible energy conversion devices and materials.

### Optimal energy-harvesting

In order to achieve optimal energy harvesting efficiency of 3D printed NMEH systems, various challenges need to be addressed, such as applications of advanced ferroelectric materials for an optimal power output of nano-energy harvesters [186]. For the biomechanical EH module, one of the potential issues is related to ultrasmall frequencies (approx. 1 Hz) of discrete and multimodal human body motion, which restricts the optimal performance of the nanodevice [187, 188]. In addition, the high operating bandwidth of the triboelectric nanogenerator is not suitable for optimal EH efficiencies [184]. Furthermore, the inconsistent input vibrations or ambient mechanical energy sources and discrepancy between applied excitation frequencies and structural resonant frequencies also impede the applications and performance of 3DP-NMEH systems. Some other challenges related to optimal structure development, materials selection, precise surface morphology, stable operation with functional units, robust packaging, appropriate power management, and adaptability with the harsh environmental circumstances are hindering the optimal efficiency of energy harvesting, and efforts should be made to solve these potential restrictions.

### MEH integration with micro/nano-systems

Several challenges related to integrating 3D printed energy harvesters with complex power management circuits and micro/nanosystems need to be addressed to commercialize the NMEHs. First, coupling with a suitable power management unit is crucial for efficiently exploiting the harvested energy by a 3DP-device. In the case of TENGs, the output is AC electrical power that cannot power DC electronics. Hence, a rectifier is required to convert AC to DC. However, the rectification efficiency can be significantly small due to impedance mismatch. Therefore, for optimal impedance matching, efficient integration, and power utilization, the application of controllers and converters is inevitable. Second, to connect powering LEDs with 3DP-NMEHs, a wireless transmitter must convert mechanical stimuli into electrical signals. Third, compliance with extreme environmental conditions such as heat, water, humidity, or critical biological or chemical elements is challenging for optimal performance. Fourth, active management and electronic components must deal with the incompatibility with the storage input and NMEHs output. Lastly, efficiently integrated energy harvesting is challenging due to the issues related to the optimal arrangements among 3DP-MEH devices, signal processing units, power management circuits, and storage.

### Commercialization challenges

Various issues hinder the commercialization of recently developed 3D printing technologies for fabricating nanogenerators that need to be overcome. The first challenge is the design and development of the optimal device structure for particular key aspects. The second issue is often related to selecting the optimal materials for the 3DP-NMEH devices to achieve optimal output under specific environmental constraints. To address the challenges mentioned above, a few questions are needed to be answered.

- it is crucial to develop precise patterns of materials using 3D printing to achieve architectural customization of the 3DP-NMEHs
- to handle multiple printable materials simultaneously and development of integrated systems comprising nanogenerators and functional units using 3D-printing [105]
- compatibility of the 3DP-NMEHs with biological, chemical, optical, and gaseous materials [189]
- optimal and stable operation of 3D-printed nano energy harvesting systems at numerous inconsistencies in biomechanical activities, natural renewable energy sources, or ambient vibrations, which constrains the use of 3DP-NMEHs.

### Other potential challenges

Electromagnetic (EMG) energy harvesters have found various mesoscale and macroscale applications such as exoskeletons and prostheses, owing to their high efficiencies and technological maturity. Nevertheless, the miniaturization of EMG devices and increasing their energy conversion efficiencies for nanoscale applications demand the development of tiny/miniature magnets and advanced magnetic materials. In addition, efforts are still needed to develop a compact configuration for miniature EMG systems by addressing the challenges of heavy gear trains and large coils.

In comparison, recently, the development of piezoelectric (PENG) and triboelectric (TENG) energy harvesters has been advanced due to enhancement in nanotechnology. However, PENGs are restricted by the lower performance of piezoelectric materials, whereas the physical mechanism behind the working of TENGs (contact electrification among various materials) is a mystery that needs to be investigated. Furthermore, the performance of TENGs can be improved by employing different working modes and device configurations.

Triboelectric nanogenerator undergoes frequent and long-term mechanical impacts to accomplish effective triboelectrification, resulting in premature device failure, output performance degradation, safety hazards, and life span loss. Further, TENGs are frequently subjected to moisture and environmental dust, causing decreased output performance and compromised durability, reliability, and robustness. Another critical issue is the inflexibility of wearable TENG devices due to their dependence on metallic electrodes. Henceforth, it is crucial to demonstrate the strategies enhancing the reliability, sustainability, durability, and safety of TENGs.

Human body movements are characterized by low frequencies even below 1 Hz. Although numerous devices have been reported to work in the low-frequency ranges comparable to human movements, the inconsistency, stochasticity, and irregularity of human body movements are rarely investigated. Therefore, it is challenging to demonstrate the energy harvesting devices adaptable to the low-frequency irregular human body motions. Furthermore, for achieving a more significant temperature gradient for TEGs and PEGs, the device configuration should be as thin as possible for better performance. In addition, the heat and biomechanical energy harvesting devices may cause mechanical discomfort resulting from the attachment of different objects with a human body and thermal discomfort due to the variation in heat exchange between sink (surroundings) and source (human body).

It is difficult to harvest significant amounts of output power from any individual MEH mechanism unless novel hybrid structures are developed to improve energy conversion coefficients. However, combining two or more energy harvesting/conversion mechanisms in one package can be effective because some physical mechanisms are naturally more compatible with each other. The potential challenges for developing hybrid NMEH devices are related to the design of device configuration, integration of functional components, and investigation of their mutual interaction for possible combinations of EH mechanisms.

Finally, the development of low-power electronics is a crucial aspect that drives the enhancement of NMEHs. Recently, the advancements in chip technology and integrated power technology have revolutionized the achievement of optimal energy harvesting capabilities and power management of electronics. Therefore, perhaps the sensors and actuators in biomechanical applications can be powered by microwatt power gadgets in the future. However, to accomplish the target, it is inevitable to develop load electronics with lower energy consumption.

## Sustainable application of plastic waste produced during COVID-19

**Fig. 18:**
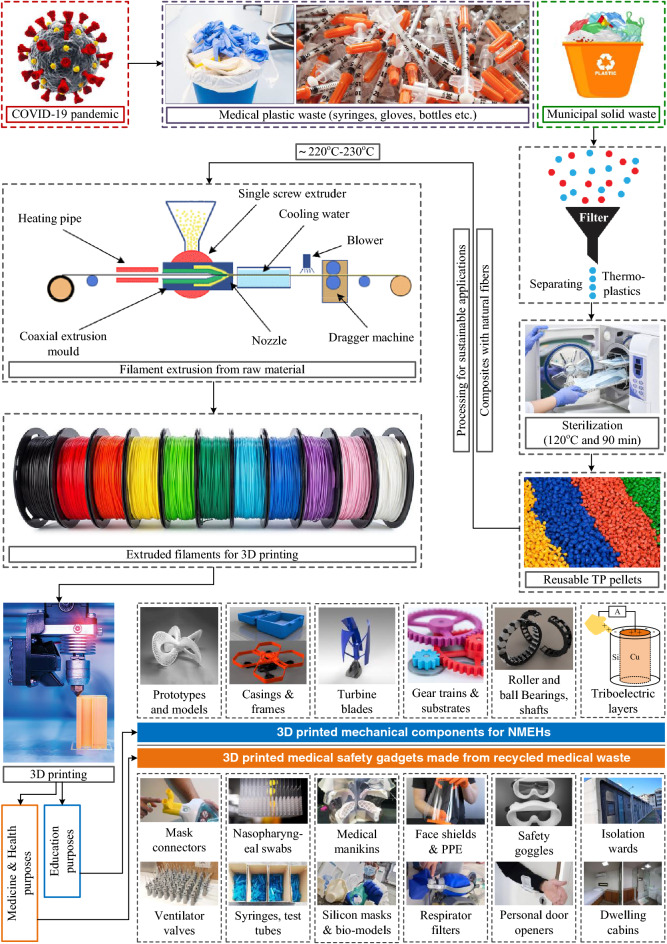
Proposal for sustainable application of plastic waste produced during COVID-19 pandemic

The COVID-19 pandemic posed major challenges related to the significantly large amount of medical plastic waste. According to a study [190], during the pandemic in China, the amount of municipal solid waste in various cities of Hubei Province was reduced by 30%, whereas the yield of medical waste increased dramatically (by 370% with a major proportion of plastic). In this situation, incineration and steam sterilization were proposed to be the most suitable waste treatment methods [190] for hazardous medical waste. However, another challenge is that the waste surge created during the pandemic significantly surpassed the overall treatment capacity. Therefore, in this study, we propose a novel sustainable solution to medical plastic waste. Most medical products are made of thermoplastics, such as polypropylene which can be melted at elevated temperatures and reformed into raw material for recycling. Hence, the thermoplastics can be sterilized and extruded for making filaments that can be used for making 3D printed components such as turbine blades/rotors, substrates, casings, frames, triboelectric films, structural members, and mechanical components (plastic gears, bearings, prototypes) of energy harvesting mechanisms (Fig. 18). In this way, the waste material can be used as a building block for small-scale sustainable energy generation systems. Moreover, the recycled sterilized raw material can also be used for 3D printing of isolation wards, packaging stuff, valves, medical face shields, door openers, training kits, test tubes, and many other components for medical and educational purposes (Fig. 18).

## Recommendations for future research

**Fig. 19:**
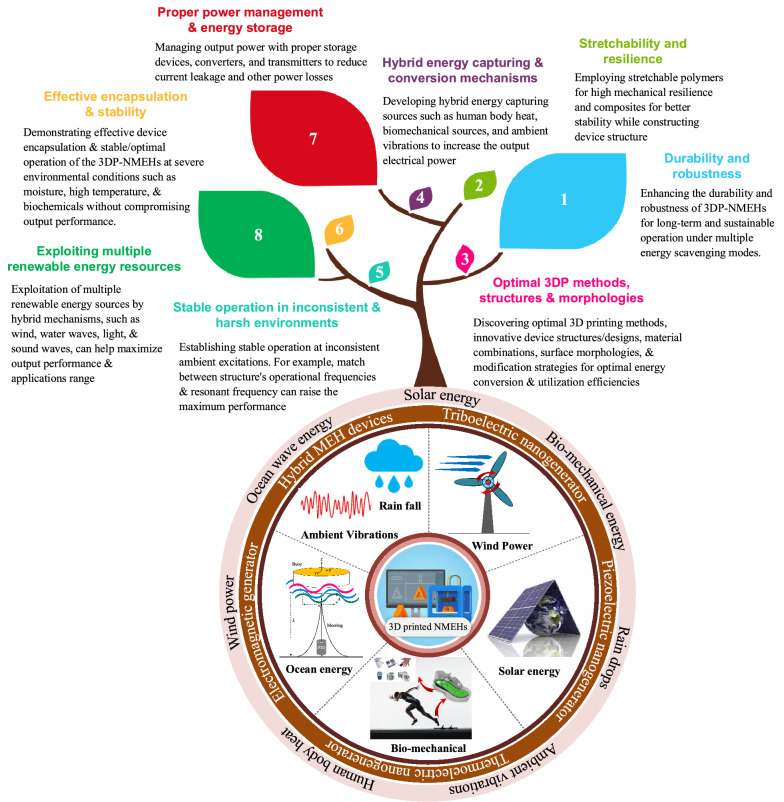
Recommendations for future research to enhance 3DP-NMEH systems

Some recommendations were extracted from a comprehensive review of the recent trends of 3DP-NMEHs, as shown in Fig. 19.

To enhance 3DP-NMEH systems, future research should be highly appreciated for,

I. Enhancing the durability and robustness of 3D printed nano MEH systems for long-term and sustainable operation of the nanodevices under multiple energy scavenging modes. For instance, triboelectric devices’ output performance and durability are augmented by combining contact separation and sliding working modes in a single device structure.
II. Employing stretchable polymers for high mechanical resilience and composites for better stability while constructing device structure.
III. Discovering optimal 3D printing methods, innovative device structures or designs, material combinations, surface morphologies, and modification strategies to maximize energy conversion and utilization efficiencies.
IV. Developing hybrid energy capturing sources such as human body heat, biomechanical sources, and ambient vibrations to increase the output electrical power.
V. Establishing stable operation at inconsistent ambient excitations or vibrational sources. For example, minimizing the variation between the structure's operational frequencies and resonant frequency can raise the maximum performance.
VI. Demonstrating effective device encapsulation and stable/optimal operation of the 3DP-NMEHs at severe environmental conditions such as moisture, high temperature, and biochemicals without compromising output performance. For instance, direct contact with bodily fluids can damage the structure and performance of in vivo devices. Moreover, for implementing an NMEH device in salty seawater for blue energy harvesting, the significance of using appropriate materials for encapsulation should not be neglected.
VII. Managing output power with proper storage devices, converters, and transmitters to reduce current leakage and other power losses.
VIII. Developing strategies to exploit multiple renewable energy sources by hybrid mechanisms, such as harvesting energy from wind, water waves, light, and sound waves through unique structures and vast combinations of materials, can help maximize the output performance and extend the range of applications.

## Summary

The recent advancements, categorization, emerging applications, challenges, and future perspectives of 3D printed nanomechanical energy harvesting systems are discussed in this focused review. The primary renewable energy sources for 3DP-NMEHs are wind, solar PV, ocean waves, railway vibrations, raindrops, biomechanical energy, and ambient vibrations. The energy conversion devices used in 3DP-NMEHs can be classified as electromagnetic, piezoelectric, triboelectric, thermoelectric, and pyroelectric generators.

Regarding the 3DP-NMEHs research contribution, China and the USA are the most significant leading countries with more than 75% contributions in enhancing 3DP-NMEHs. In contrast, the most influential institutions publishing the most significant publications in the 3DP-NMEH research field are the Georgia Institute of Technology, University of California Berkeley, Nanyang Technological University, Chinese Academy of Sciences, and Univ Texas EL PASO.

Extensive literature review revealed that most of the nano-scaled 3D printed EMG, PENG, and TENG devices achieved an output power within the range of 0.5‒32 mW, 0.0002‒45.6 mW, and 0.3‒4.67 mW, respectively, which could be appropriately selected for varying power requirements for a wide range of applications. The emerging applications of 3DP-NMEHs are sustainable/portable energy supplies for self-powered wireless sensors, actuators, inertial sensors, biomedical health monitoring sensors, pollution degradation electro-Fenton systems, electronic textiles, ramie fiber degumming, wastewater treatment, noise cancellation, driver habits monitoring, EH from sweat-eating bacteria, robotic grippers, dust-adsorption systems, wearable electronics and speech recognition. Some other interesting applications are alcohol detection, weather sensors, sensors in railway tunnels, and structural health or crack monitoring.

The potential challenges confronted by 3DP-NMEHs are related to limited 3D printing techniques, insufficient printable materials, material functionalization, commercialization, optimal energy harvesting, integration with micro/nano-systems, complex power management, encapsulation for biomedical implants, inadequate knowledge of hybrid nano-MEHs and ultra-low frequencies of human body motions, durability/reliability of the device under extreme environmental conditions, and mismatch between the input excitation frequencies and resonance bandwidth of most of the nanogenerators.

For future research, significant efforts are required to.

I. Discover optimal 3D printing methods, innovative designs, material combinations, and surface modification strategies to maximize energy conversion and efficiency.
II. Develop strategies for integrating NMEHs and complex power management circuits to reduce current leakage and other power losses.
III. Maintain stable operation at inconsistent ambient excitations.
IV. Exploit multiple renewable energy resources by hybrid 3DP-NMEH mechanisms.
V. Demonstrate effective device encapsulation and sustainable operation of the 3DP-NMEHs at severe environmental conditions.
VI. Discover new, printable, and biocompatible piezoelectric and thermoelectric materials for optimal energy harvesting.
VII. Establish strategies for multi-material printing for integrated manufacturing of NMEHs and electronic circuits,
VIII. Design the flexible energy conversion devices for the curved surface of human skin.
IX. Design specialty printer head for inkjet printing of various functional ceramic materials for piezoelectric devices.
X. Determine the 3DP materials and strategies to develop coils and magnets for miniaturizing electromagnetic generators.
XI. Study the design of structural configuration and the interaction between different mechanisms to achieve hybridization of different energy conversion technologies.

## Supplementary Information

**Additional file 1.**

## Data Availability

The additional data is provided in the supplementary file with this manuscript.
