# Supplementary material for: Additively manufactured nano-mechanical energy harvesting systems: advancements, potential applications, challenges and future perspectives
Source: Nano Converg. 2021 Dec 1;8:37. doi: 10.1186/s40580-021-00289-0 (PMC8633623; doi:10.1186/s40580-021-00289-0)
Supplement: Supplementary file 1 — Additional file 1. [file 40580_2021_289_MOESM1_ESM.docx]

**Supplementary file**

**Additively manufactured Nano-mechanical energy harvesting systems: Advancements, potential applications, challenges and future perspectives**

**Note:**

The research papers cited in **Table S1**, **Table S2**, **Table S3**, **Table S4**, **Table S5**, **Table S6** and **Table S7** are given in this supplementary file

**Table S1.** Features and characterization of some previously developed nano-systems to harvest renewable energy from natural resources (wind, ocean and solar resources)^[[1]](#footnote-1)^

| **Energy conversion** | **Mechanism** | **Input** | **Output** | **Advantages (**$\boldsymbol{\uparrow}$**)** **and** **disadvantages (↓)** | **Ref.** |
| --- | --- | --- | --- | --- | --- |
| Electromagnetic generators (EMG) | Portable wind energy harvester based on S-rotor and H-rotor | 5-12 m/s | 108 mW, 23.2% | ($\uparrow$) powers the monitoring sensors in railway tunnels  ($\uparrow$) uses hybrid S-rotor and H-rotor | [1] |
|  | Double-Skin Façade system for harvesting wind energy | 3-8 m/s | 1110 W/m^2^ | ($\uparrow$) low turbulence and uniform flow due to cavity  (↑) provides a wide range of angles for incident wind | [2] |
|  | Galloping, vortex shedding, flutter and aerodynamic instability | 2-6 m/s | 1 W | (↑) based on wake galloping  (↑) a simpler mechanism for structural health monitoring system  (↑) powers wireless sensors | [3] |
| Piezoelectric nanogenerators (PENG) | The flutter of a flexible piezoelectric membrane | 9 m/s | 5 mW/cm^3^ | (↑) simple inverted flag orientation  (↑) Self-aligning capability  (↑) can operate in low-speed regimes | [4] |
|  | Vortex-induced vibration-based piezoelectric EH | 1-1.4 m/s | 0.6 mW | (↑) facilitates Y-shaped attachments on bluff body  (↑) provides an enhanced energy harvesting efficiency | [5] |
|  | MEH composed of permanent magnets, rotor, piezoelectric stack and flexure mechanism | 100 rpm | 0.2 mW | (↑) simple and compact design  (↑) optimal performance with larger power output | [6] |
| Pyroelectric (PEG)/ Thermoelectric generators (TEG) | Flexible vortex generator or turbulator | 1-25 m/s | 3 $\mu$W/cm^2^ | (↑) Flexible structure with un-interrupted energy output  (↓) possesses low pyroelectric coefficient | [7] |
|  | Harvesting solar and wind energies using thermal  oscillations through sustainable PEG | 2.5-5.3 m/s | 421 $\mu$W/cm^3^ | (↑) provides high power density  (↓) power density depends on the intensity of the solar irradiations and wind speed | [8] |
| Triboelectric nanogenerators (TENG) | A rotary TENG based on mechanical deformation of multiple plates | 15 m/s | 39 W/m^2^ | (↑) facilitates the application of polymer nanowires  (↑) can be used as a self-powered wind speed sensor | [9] |
|  | TENG-based windmill composed of nanopillar-array architectured layers | 14-15 m/s | 568 V, 26 μA | (↑) simple and cheap fabrication  (↑) high output and optimal performance  (↑) high stability | [10] |
|  | Pendulum-based TENG using a pendulum structure with high energy conversion efficiency | 2 m/s, 2 Hz | 56 V | (↑) superior durability  (↑) ultrahigh sensitivity  (↑) long-time operation  (↑) energy harvesting from wave and wind | [11] |

**Table S2.** Triboelectric nanogenerator based 3D printed energy harvesting devices, their output energy capacities and applications^[[2]](#footnote-2)^

| **Ref.** | **Energy harvesting devices** | **Source of excitation** | **Excitations** | **Materials** | **Output** | **Applications** |
| --- | --- | --- | --- | --- | --- | --- |
| [12] | Wrist-wearable TENG device | Human wrist-motions | ≤5 Hz | ABS, PLA | 0.118 mW/cm^3^ | Wearable electronic devices, self-powered healthcare monitoring sensors |
| [13] | Bidirectional gear transmission based TENG | Motion of human foot | 3.5 Hz | PLA | 4 mW | LEDs, thermometers, low-power devices |
| [14] | Elastic TENG based self-powered electro-fenton system | Reciprocation by hand | 2–5 Hz | Acrylic | 1.95 W/m^2^ | Sustainable removal of methylene blue (MB) emissions, LED bulbs |
| [15] | Hybrid coaxial TENG | Rotary motion | 100-400 rpm | ABS, acrylic | 846.4 $\mu$W | LEDs, small toys, sensors |
| [16] | Wind-driven hybrid TENG nanogenerator | Slow speed wind | 6 m/s | PLA | 245 mW | Subway tunnel, electronic gadgets, wireless sensor nodes, LED screen |
| [17] | Freestanding kinetic-impact-based TENG | Human motions | 5 Hz | PLA | 102.29 mW | Thermo-hygrometers, LEDs, smartphones, smartwatches, temperature sensors |
| [18] | Flexible TENG for vibration energy harvesting | Vibrations | 6 Hz | Acrylic | 608.5 mW/m^2^ | Portable and wearable sensors |
| [19] | 3D-printed silicone-Cu fiber-based TENG | Human motion | ≤5 Hz | Si elastomer | 31.39 mW/m^2^ | Sensors, energy harvesting, LEDs, biomechanical applications |
| [20] | Integrated flywheel & spiral spring TENG | Human foot motion | ≤5 Hz | PLA | 38.4 mJ | LEDs, commercial thermometer, small electronic devices |
| [21] | Low frequency resonant TENG nanogenerator | Manual vibrations | 18 Hz | ABS | 2.61 mW | Vibration sensors, portable and wearable electronic devices, recharging batteries |
| [22] | Novel sweep-type TENG | Rotary motion | 1.2 m/s | PLA | 400V, 15 μA | Thermometer, LEDs, driver habits-monitoring, road conditions analysis |
| [23] | Mechanical frequency regulator based TENG | Human and windmill | 10–50 Hz | PLA | 17V, 6.5 mA | Wireless node sensors |
| [24] | Water droplet vibrations based TENGs | Vibrations | 1 to 30 Hz | ITO glass | 7.55 μW | Self-powered electronic systems |
| [25] | Origami-tessellation-based TENG | Ambient excitations | 3 to 16 Hz | Nylon | 26.16 μW | Energy harvesting on road pavement |
| [26] | Galloping TENG based on two flexible beams | Wind energy | 1.4 to 6 m/s | ABS, PET | 200V, 7 μA | Outdoor electric devices, LEDs |
| [27] | Direction-switchable TENG | Human joint motions | 5 to 15 cm/s | PLA | 5V, 10 μA | Portable self-powered electronic devices |
| [28] | Rotary cam-based TENG | Rotary motion | 300-1000 rpm | PLA | 3.5 mW | LEDs, commercial & industrial applications |
| [10] | Nanopillar-array architectured TENG | Wind energy | 14–15 m/s | PLA | 568V, 25.6 μA | Wind energy harvesting |

EMG electromagnetic generator

TENG triboelectric nanogenerator

ABS acrylonitrile poly-butadiene styrene

PLA polylactic acid

ITO Indium tin oxide

**Table S3.** Piezoelectric nanogenerator based 3D printed energy harvesting devices, their output energy capacities and applications^[[3]](#footnote-3)^

| **Ref.** | **Energy harvesting devices** | **Source of excitation** | **Excitations** | **Materials** | **Output** | **Applications** |
| --- | --- | --- | --- | --- | --- | --- |
| [29] | Piezoelectric ceramics for MEH | Vibrations | – | Photocurable resin | 0.301 V | Energy focusing and ultrasonic  sensing |
| [30] | Piezoelectric BNNTs nanocomposites | Biomechanical energy | 10 Hz | Photocurable resin | 24 mV/kPa | Self-powered conformal sensors, haptic sensing of robotic hand |
| [31] | 3D-printed PVDF-TrFE piezoelectric film | Finger and wrist joints | 0.5–4Hz | PVDF-TrFE | 73.5 V | External stress stimulation, self-powered tactile sensors, artificial skin |
| [32] | Stretchable kirigami piezoelectric nanogenerator | Vibrations from magnetic shaker | 5 Hz | piezoelectric  ink | 1.4 μW/cm^2^ | Self-powered gait sensor |
| [33] | Stretchable piezoelectric nanogenerator | Vibrations from magnetic shaker | 5 Hz | 3D printable ink | 0.29 V | Wearable electronic systems, self-powered  body motion sensor |
| [34] | 3DAIS | 3D vibration, rotation & human motion | 2.5 Hz | Acrylic | 0.19 µW | Multi-axis rotation and acceleration inertial sensing, telemedicine applications |
| [35] | Stiffness-tunable soft  robotic gripper | Finger bending | 1 mm/s | FLX9760, RGD8530 | 3 V | Anthropomorphic grippers |
| [36] | Ceramic-polymer composite | Universal testing machine | 100 Hz | Grid-composite | 270 mV | Flexible electronics application, force sensor applications |

BNNTs Boron nitride nanotubes

PVDF-TrFE Poly (vinylidene fluoride-co-trifluoroethylene)

3DAIS 3D activity inertial sensor

**Table S4.** Thermoelectric generator based 3D printed energy harvesting devices, their output energy capacities and applications^[[4]](#footnote-4)^

| **Ref.** | **Energy harvesting devices** | **Source of excitation** | $\boldsymbol{\Delta T}$ | **Materials** | **Performance** | **Applications** |
| --- | --- | --- | --- | --- | --- | --- |
| [37] | Flexible thermoelectric power generator | Electric heater | 30 K | TE materials | 80 mV | Wearable self-powered electronics |
| [38] | Segmented thermoelectric generators | Ceramic heater | 236 °C | BiSbTe-based viscoelastic inks | 8.7% | Self-powered electronic sensors |
| [39] | Conformal cylindrical thermoelectric generators | Hot water flowing through alumina pipe | 39 °C | Bi_2_Te_3_-based inks | 1.62 mW | Power generation from waste heat |
| [40] | 3D printed SnSe thermoelectric generators | Thermoelectric tester | 772 K | Tin selenide (SnSe) | 20 µW | Solar cell applications |
| [41] | Flexible and stretchable organic thermoelectric device | Heating controller | 75 K | Polyurethane/CNT nanocomposites | 19.8 ± 0.2 µV/K | Potential large-scale energy harvesting applications |
| [42] | Shape-controllable thermoelectric devices | Heating rod | 54.6 K | Bi_2_Te_3_/(PVP) composites | 0.68 mW | Harvesting energy from waste heat |
| [43] | Self-healing & stretchable  3D-printed TE device | Body temperature | 7 K | PEDOT: PSS | 12.2 nW | Flexible and wearable electronics and sensors |
| [44] | Thick printed TE  generator | Microelectronic heat sink | 40 °C | Bi_2_Te_3_-based TE ingot | 10 W/cm^2^ | Microelectronic applications |

SnSe Tin selenide

CNT carbon nanotubes

PVP polyvinylpyrrolidone

TE thermoelectric

PEDOT: PSS poly(3,4-ethylenedioxythiophene) doped with polystyrene sulfonate

**Table S5.** TENG, PENG, TEG and EMG based 3D printed hybrid MEH devices, their output energy capacities and applications^[[5]](#footnote-5)^

| **Ref.** | **Energy harvesting devices** | **Source of excitation** | **Excitations** | **Materials** | **Output** | **Applications** |
| --- | --- | --- | --- | --- | --- | --- |
| [12] | Hybrid EMG-TENG wrist-wearable device | Human wrist-motions | 5 Hz | ABS, PLA | 0.118 mW/cm^3^ | Wearable electronic devices, self-powered healthcare monitoring sensors |
| [34] | Hybrid EMG-TENG-PENG 3DAIS device | 3D vibration, rotation & human motion | 2.5 Hz | Acrylic | 0.19 µW | Multi-axis acceleration & rotation inertial sensing, telemedicine applications |
| [16] | Hybrid EMG-TENG wind-driven nanogenerator | Slow speed wind | 6 m/s | PLA | 245 mW | Subway tunnel, electronic gadgets, wireless sensor nodes, LED screen |
| [21] | Hybrid EMG-TENG device resonating at low frequency | Manual vibrations | 18 Hz | ABS | 2.61 mW | Vibration sensors, portable and wearable electronic devices, recharging batteries |
| [45] | Hybrid TENG-EMG-PENG  energy harvester | Hybrid step-servo  motor | 45 rpm (0.75 Hz) | ABS | 712μW, 31 mW, 6.4 μW | Self-powered sensing, harvest rotational mechanical energy |
| [46] | Solar & electromagnetic Energy harvesting System | Solar irradiance | 100 mW/cm^2^ | PLA | 93 mW | Internet-of-Things Wireless Sensors |
| [47] | 3D printed miniature EMG device driven by airflow | Wind energy, wind tunnel | – | ABS | 0.305 W | HVAC (heating, ventilating and air conditions) ventilation exhaust systems |
| [48] | Hybrid EMG-TENG rotating gyro structured blue EH | Blue energy | 1.2 to 2.3 Hz | White resin | 14.9 mW (EMG) 4.1 μW (TENG) | Self-powered & self-functional tracking system |
| [49] | Ship-shaped hybridized nanogenerator (SHNG) | Blue energy (linear motor) | 2 Hz | PLA | 800 µW (TENG) 9 mW (EMG) | Seawater self-desalination and self-powered positioning |

EMG electromagnetic generator

TENG triboelectric nanogenerator

PENG piezoelectric nanogenerator

3DAIS 3D activity inertial sensor

ABS acrylonitrile poly-butadiene styrene

PLA polylactic acid

EH Energy harvester

**Table S6.** Optimal 3D printing methods, printing parameters and 3D printers involved in fabricating novel structures of 3DP-NMEHs^[[6]](#footnote-6)^

| **Sr.** | **Structure/shape** | **Printing approach** | **3D printer company** | **Printing parameters** | **Applications** | **Ref.** |
| --- | --- | --- | --- | --- | --- | --- |
| 1 | Hierarchical and porous  structures | FDM | HTS-300, Fochif Tech.,  pressure-controlled direct ink printer | Deposition speed of 2.8 mm s^-1^, extrusion  speed of 0.008 mm s^-1^, filament diameter of 0.85 mm and micro-nozzle diameter of 0.80 mm | Wearable electronics | [50] |
| 2 | Circular-shaped structures | FDM | Shining, Einstart-p,  3D printer | Uniform material extrusion from needle | Voiceprint recognition sensor | [51] |
| 3 | Square-shaped structures | FDM | 30M Hyrel 3D, USA 3D printer | Nozzle inner diameter of 0.5 mm | Self-healing/ stretchable  conductor | [52] |
| 4 | Cubical shape | Hybrid UV based  3D printing | 3D printer equipped with automatic UV curing, pressure-injection, and ink extrusion along with precision positioning platform | UV-based curing and printing precision of 1 μm | Ultra-flexible 3D printed TENG | [53] |
| 5 | Cylindrical structures | FDM | Makerbot Industry, USA,  Replicator 2X 3D printer | Printing speed 90 mms^-1^ with plate temperature of 110 $℃$, using a raft to improve the adhesion  between the plate and 3D printed parts | Noise-canceling | [54] |
| 6 | Hollow circular-shaped tubes | FDM | **‒** | 3D-printed circular tube of 1.1 cm inner dia and 1.2 cm outer dia | Human biomechanical  energy harvesting | [12] |
| 7 | Lamellar  porous constructions | DIW | **‒** | An 840 μm dia cylindrical nozzle to print CNF ink through DIW printer | Multifunctional sensors | [55] |
| 8 | Biomimetic-villus shaped  structure | DLP | Master Plus J 845 DLP printer from Carima, Korea | **‒** | Dust filter | [56] |
| 9 | Grating disk-like structure | FDM, SLM | ProX DMP 320 from 3D Systems,  metal 3D printer | 3D printing in argon gas with a 245 W laser, with 60 μm layer thickness, 82 μm side step, and 1250 mm s^-1^ mark speed | Sustainable energy harvesting | [57] |
| 10 | Sponge | FDM | Z300, Beijing Huitianwei  Technology Co., Ltd, China, 3D FDM printer | Nozzle size of approx. 10 μm | Energy harvesting applications | [58] |
| 11 | Zigzag design | FDM | FDM printer Z300, Beijing  Huitianwei Technology Co., Ltd, China | Material extrusion with 0.1 mm layer height | Mechanical energy harvesting | [59] |
| 12 | Hierarchical morphological  structures | DIW | **‒** | Parallel printing direction with 0.2 mm tip diameter | Mechanical energy harvesting | [18] |

FDM fused deposition modeling

DIW direct ink writing

SLM selective laser melting

UV ultraviolet

**Table S7.** Benefits and challenges related to 3D-printing of the nano MEH systems^[[7]](#footnote-7)^

| **Benefits** | The accurate pattern-making ability for architectural customization of the nanogenerators. The tiny structured patterns allow easy implementation and mechanical resilience to electronics. | [60] |
| --- | --- | --- |
|  | Lower power consumption and environmental impact. Facilitates long-term sustained production. | [61] |
|  | Provides faster speed and high-fabrication compatibility | [62] |
|  | Less human intervention is required in the printing of the parts and post-treatment | [63] |
|  | Abatement of waste materials and overall material usage | [51] |
|  | 3D printing is a safe, sensitive, and flexible fabrication facility | [53] |
|  | Excellent chemical attributes characterize the printed objects | [52], [55] |
|  | FDM printing gives heat resistance and good mechanical strength to the prototypes | [14], [51] |
|  | Facilitates easy and manual removal of the supporting elements, i.e., water-soluble wax at the end of the printing process | [18] |
|  | Fast solidification of the printed components on various substrates | [58] |
|  | Exceptional printing accuracy and resolution | [64] |
| **Limitations** | Difficult integration of functional polymers | [65] |
|  | To achieve and maintain the tiny gap required between triboelectric polymer surfaces is challenging | [65] |
|  | The formation of precise macro/nano dimensional architectures is yet to be studied | [65] |
|  | The combination of various materials for 3D printed TENGs is problematic | [12] |
|  | The development of integrated systems comprising of nanogenerators, and functional devices and simultaneous handling of multiple printing materials is still challenging | [65] |
|  | 3D printed nano MEH systems are needed to be biocompatible and integrated with biological tissues | [65] |
|  | End-of-life recyclability/biodegradability of the 3D-printed components is a critical concern to reduce impacts on the environment and the human body | [65] |
|  | For complex models, the printing time is often high | [56] |
|  | Digital light processing (DLP) demands more light sources, for instance, arc lamps during printing | [56] |
|  | A liquid crystal display is employed at the entire 3D-printed deposit during a single layer of the DLP | [56] |
|  | In 3D printing of fabrics, it is challenging to develop appropriate CAD modeling in order to facilitate the simulation of the draping of the textile across a curved surface | [66] |
|  | 3D-printed textiles are not strong enough and tend to break easily due to the conventional textiles' lesser flexibility. Hence, they are not very suitable for day to day textile applications | [67] |

**References**

1. H. Pan, H. Li, T. Zhang, A. A. Laghari, Z. Zhang, Y. Yuan, and B. Qian, Energy Convers. Manag. **196**, 56 (2019).

2. S. Hassanli, G. Hu, K. C. S. Kwok, and D. F. Fletcher, J. Wind Eng. Ind. Aerodyn. **167**, 114 (2017).

3. H.-J. Jung, S.-W. Lee, and D.-D. Jang, IEEE Trans. Magn. **45**, 4376 (2009).

4. S. Orrego, K. Shoele, A. Ruas, K. Doran, B. Caggiano, R. Mittal, and S. H. Kang, Appl. Energy **194**, 212 (2017).

5. J. Wang, S. Zhou, Z. Zhang, and D. Yurchenko, Energy Convers. Manag. **181**, 645 (2019).

6. Z. Wu and Q. Xu, in *2018 IEEE Int. Conf. Mechatronics Autom.* (IEEE, 2018), pp. 1722–1727.

7. M. H. Raouadi and O. Touayar, Sensors Actuators A Phys. **273**, 42 (2018).

8. S. H. Krishnan, D. Ezhilarasi, G. Uma, and M. Umapathy, IEEE Trans. Sustain. Energy **5**, 73 (2013).

9. Y. Xie, S. Wang, L. Lin, Q. Jing, Z.-H. Lin, S. Niu, Z. Wu, and Z. L. Wang, ACS Nano **7**, 7119 (2013).

10. B. Dudem, N. D. Huynh, W. Kim, D. H. Kim, H. J. Hwang, D. Choi, and J. S. Yu, Nano Energy **42**, 269 (2017).

11. Z. Lin, B. Zhang, H. Guo, Z. Wu, H. Zou, J. Yang, and Z. L. Wang, Nano Energy **64**, 103908 (2019).

12. P. Maharjan, H. Cho, M. S. Rasel, M. Salauddin, and J. Y. Park, Nano Energy **53**, 213 (2018).

13. X. Lu, Y. Xu, G. Qiao, Q. Gao, X. Zhang, T. Cheng, and Z. L. Wang, Nano Energy 104726 (2020).

14. M. Tian, D. Zhang, M. Wang, Y. Zhu, C. Chen, Y. Chen, T. Jiang, and S. Gao, Nano Energy 104908 (2020).

15. D. Lee and D. Kim, Nano Energy **71**, 104599 (2020).

16. M. T. Rahman, M. Salauddin, and J. Y. Park, in *2019 20th Int. Conf. Solid-State Sensors, Actuators Microsystems Eurosensors XXXIII (TRANSDUCERS EUROSENSORS XXXIII)* (IEEE, 2019), pp. 1443–1446.

17. M. T. Rahman, S. M. S. Rana, M. Salauddin, P. Maharjan, T. Bhatta, H. Kim, H. Cho, and J. Y. Park, Appl. Energy **279**, 115799 (2020).

18. H. Li, R. Li, X. Fang, H. Jiang, X. Ding, B. Tang, G. Zhou, R. Zhou, and Y. Tang, Nano Energy **58**, 447 (2019).

19. Y. Tong, Z. Feng, J. Kim, J. L. Robertson, X. Jia, and B. N. Johnson, Nano Energy 104973 (2020).

20. W. Yang, Y. Wang, Y. Li, J. Wang, T. Cheng, and Z. L. Wang, Nano Energy **66**, 104104 (2019).

21. M. Salauddin, R. M. Toyabur, P. Maharjan, M. S. Rasel, H. Cho, and J. Y. Park, Nano Energy **66**, 104122 (2019).

22. Z. Xie, Z. Zeng, Y. Wang, W. Yang, Y. Xu, X. Lu, T. Cheng, H. Zhao, and Z. L. Wang, Nano Energy **68**, 104360 (2020).

23. D. Bhatia, J. Lee, H. J. Hwang, J. M. Baik, S. Kim, and D. Choi, Adv. Energy Mater. **8**, 1702667 (2018).

24. J. Ding, W.-Q. Tao, and S.-K. Fan, Nano Energy **70**, 104473 (2020).

25. H. Zhang, C. Yang, Y. Yu, Y. Zhou, L. Quan, S. Dong, and J. Luo, Nano Energy **78**, 105177 (2020).

26. L. Zhang, B. Meng, Y. Xia, Z. Deng, H. Dai, P. Hagedorn, Z. Peng, and L. Wang, Nano Energy **70**, 104477 (2020).

27. S. Cho, Y. Yun, S. Jang, Y. Ra, J. H. Choi, H. J. Hwang, D. Choi, and D. Choi, Nano Energy **71**, 104584 (2020).

28. Y. Lee, W. Kim, D. Bhatia, H. J. Hwang, S. Lee, and D. Choi, Nano Energy **38**, 326 (2017).

29. Z. Chen, X. Song, L. Lei, X. Chen, C. Fei, C. T. Chiu, X. Qian, T. Ma, Y. Yang, and K. Shung, Nano Energy **27**, 78 (2016).

30. J. Zhang, S. Ye, H. Liu, X. Chen, X. Chen, B. Li, W. Tang, Q. Meng, P. Ding, and H. Tian, Nano Energy **77**, 105300 (2020).

31. X. Yuan, X. Gao, X. Shen, J. Yang, Z. Li, and S. Dong, Nano Energy **85**, 105985 (2021).

32. X. Zhou, K. Parida, O. Halevi, Y. Liu, J. Xiong, S. Magdassi, and P. S. Lee, Nano Energy **72**, 104676 (2020).

33. X. Zhou, K. Parida, O. Halevi, S. Magdassi, and P. S. Lee, Sensors **20**, 6748 (2020).

34. K. H. Koh, Q. Shi, S. Cao, D. Ma, H. Y. Tan, Z. Guo, and C. Lee, Nano Energy **56**, 651 (2019).

35. M. Xie, M. Zhu, Z. Yang, S. Okada, and S. Kawamura, Nano Energy **79**, 105438 (2021).

36. Z. Wang, X. Yuan, J. Yang, Y. Huan, X. Gao, Z. Li, H. Wang, and S. Dong, Nano Energy **73**, 104737 (2020).

37. N. Nandihalli, C.-J. Liu, and T. Mori, Nano Energy 105186 (2020).

38. S. E. Yang, F. Kim, F. Ejaz, G. S. Lee, H. Ju, S. Choo, J. Lee, G. Kim, S. Jung, and S. Ahn, Nano Energy **81**, 105638 (2021).

39. F. Kim, B. Kwon, Y. Eom, J. E. Lee, S. Park, S. Jo, S. H. Park, B.-S. Kim, H. J. Im, and M. H. Lee, Nat. Energy **3**, 301 (2018).

40. M. R. Burton, S. Mehraban, D. Beynon, J. McGettrick, T. Watson, N. P. Lavery, and M. J. Carnie, Adv. Energy Mater. **9**, 1900201 (2019).

41. L. Tzounis, M. Petousis, S. Grammatikos, and N. Vidakis, Materials (Basel). **13**, 2879 (2020).

42. N. Su, P. Zhu, Y. Pan, F. Li, and B. Li, Energy **195**, 116892 (2020).

43. S. Kee, M. A. Haque, D. Corzo, H. N. Alshareef, and D. Baran, Adv. Funct. Mater. **29**, 1905426 (2019).

44. C. Navone, M. Soulier, J. Testard, J. Simon, and T. Caroff, J. Electron. Mater. **40**, 789 (2011).

45. T. Ma, Q. Gao, Y. Li, Z. Wang, X. Lu, and T. Cheng, Adv. Eng. Mater. **22**, 1900872 (2020).

46. J. Bito, R. Bahr, J. G. Hester, S. A. Nauroze, A. Georgiadis, and M. M. Tentzeris, IEEE Trans. Microw. Theory Tech. **65**, 1831 (2017).

47. N. Han, D. Zhao, J. U. Schluter, E. S. Goh, H. Zhao, and X. Jin, Appl. Energy **178**, 672 (2016).

48. L. Gao, S. Lu, W. Xie, X. Chen, L. Wu, T. Wang, A. Wang, C. Yue, D. Tong, and W. Lei, Nano Energy 104684 (2020).

49. H. Wang, Q. Zhu, Z. Ding, Z. Li, H. Zheng, J. Fu, C. Diao, X. Zhang, J. Tian, and Y. Zi, Nano Energy **57**, 616 (2019).

50. S. Chen, Y. Song, D. Ding, Z. Ling, and F. Xu, Adv. Funct. Mater. **28**, 1802547 (2018).

51. R. Guo, H. Zhang, Z. Pei, S. Yang, C. Ge, S. Sang, and R. Hao, Adv. Eng. Mater. **22**, 1901560 (2020).

52. K. Parida, G. Thangavel, G. Cai, X. Zhou, S. Park, J. Xiong, and P. S. Lee, Nat. Commun. **10**, 1 (2019).

53. B. Chen, W. Tang, T. Jiang, L. Zhu, X. Chen, C. He, L. Xu, H. Guo, P. Lin, and D. Li, Nano Energy **45**, 380 (2018).

54. J. P. Lee, B. U. Ye, K. N. Kim, J. W. Lee, W. J. Choi, and J. M. Baik, Nano Energy **38**, 377 (2017).

55. C. Qian, L. Li, M. Gao, H. Yang, Z. Cai, B. Chen, Z. Xiang, Z. Zhang, and Y. Song, Nano Energy **63**, 103885 (2019).

56. H.-J. Yoon, D.-H. Kim, W. Seung, U. Khan, T. Y. Kim, T. Kim, and S.-W. Kim, Nano Energy **63**, 103857 (2019).

57. M.-L. Seol, R. Ivaškevičiūtė, M. A. Ciappesoni, F. V Thompson, D.-I. Moon, S. J. Kim, S. J. Kim, J.-W. Han, and M. Meyyappan, Nano Energy **52**, 271 (2018).

58. S. He, Z. Yu, H. Zhou, Z. Huang, Y. Zhang, Y. Li, J. Li, Y. Wang, and D. Li, Nano Energy **52**, 134 (2018).

59. H. Qiao, Y. Zhang, Z. Huang, Y. Wang, D. Li, and H. Zhou, Nano Energy **50**, 126 (2018).

60. J. Wang, B. Wu, G. Liu, T. Bu, T. Guo, Y. Pang, X. Fu, J. Zhao, F. Xi, and C. Zhang, Extrem. Mech. Lett. **20**, 38 (2018).

61. C. Chen, L. Chen, Z. Wu, H. Guo, W. Yu, Z. Du, and Z. L. Wang, Mater. Today **32**, 84 (2020).

62. M. S. Rasel, P. Maharjan, M. Salauddin, M. T. Rahman, H. O. Cho, J. W. Kim, and J. Y. Park, Nano Energy **49**, 603 (2018).

63. P. Maharjan, R. M. Toyabur, and J. Y. Park, Nano Energy **46**, 383 (2018).

64. S. Tol, F. L. Degertekin, and A. Erturk, Addit. Manuf. **29**, 100780 (2019).

65. M. A. P. Mahmud, A. Zolfagharian, S. Gharaie, A. Kaynak, S. H. Farjana, A. V. Ellis, J. Chen, and A. Z. Kouzani, Adv. Energy Sustain. Res. **2**, 2000045 (2021).

66. G. A. Bingham, R. J. M. Hague, C. J. Tuck, A. C. Long, J. J. Crookston, and M. N. Sherburn, Int. J. Comput. Integr. Manuf. **20**, 96 (2007).

67. S. Gowthaman, G. S. Chidambaram, D. B. G. Rao, H. V. Subramya, and U. Chandrasekhar, J. Inst. Eng. Ser. C **99**, 435 (2018).

1. The references of the research papers cited in “Table 2” are provided in the supplementary file of this manuscript. [↑](#footnote-ref-1)
2. The references of the research papers cited in “Table 3” are provided in the supplementary file of this manuscript. [↑](#footnote-ref-2)
3. The references of the research papers cited in “Table 4” are provided in the supplementary file of this manuscript. [↑](#footnote-ref-3)
4. The references of the research papers cited in “Table 5” are provided in the supplementary file of this manuscript. [↑](#footnote-ref-4)
5. The references of the research papers cited in “Table 6” are provided in the supplementary file of this manuscript. [↑](#footnote-ref-5)
6. The references of the research papers cited in “Table 8” are provided in the supplementary file of this manuscript. [↑](#footnote-ref-6)
7. The references of the research papers cited in “Table 9” are provided in the supplementary file of this manuscript. [↑](#footnote-ref-7)
